# Molecularly engineered covalent hydrophobic interface for enhanced CO_2_ electromethanation in strong acid

**DOI:** 10.1093/nsr/nwag116

**Published:** 2026-02-24

**Authors:** Chang Zhu, Dashuai Wang, Nengji Liu, Weixiao Lin, Libin Zeng, Yaqi Chen, Wanzhen Zheng, Xianyun Peng, Guanghui Feng, Zhongjian Li, Bin Yang, Xiahan Sang, Lecheng Lei, Fei Song, Paolo Samorì, Yang Hou

**Affiliations:** Key Laboratory of Biomass Chemical Engineering of Ministry of Education, College of Chemical and Biological Engineering, Zhejiang University, Hangzhou 310027, China; Research Center of Industrial Ecology, Institute of Zhejiang University – Quzhou, Quzhou 324000, China; Key Laboratory of Biomass Chemical Engineering of Ministry of Education, College of Chemical and Biological Engineering, Zhejiang University, Hangzhou 310027, China; Research Center of Industrial Ecology, Institute of Zhejiang University – Quzhou, Quzhou 324000, China; Key Laboratory of Biomass Chemical Engineering of Ministry of Education, College of Chemical and Biological Engineering, Zhejiang University, Hangzhou 310027, China; Shanghai Synchrotron Radiation Facility, Shanghai Advanced Research Institute, Chinese Academy of Sciences, Shanghai 201800, China; State Key Laboratory of Advanced Technology for Materials Synthesis and Processing, Wuhan University of Technology, Wuhan 430070, China; Key Laboratory of Biomass Chemical Engineering of Ministry of Education, College of Chemical and Biological Engineering, Zhejiang University, Hangzhou 310027, China; Research Center of Industrial Ecology, Institute of Zhejiang University – Quzhou, Quzhou 324000, China; Key Laboratory of Biomass Chemical Engineering of Ministry of Education, College of Chemical and Biological Engineering, Zhejiang University, Hangzhou 310027, China; Key Laboratory of Biomass Chemical Engineering of Ministry of Education, College of Chemical and Biological Engineering, Zhejiang University, Hangzhou 310027, China; Key Laboratory of Biomass Chemical Engineering of Ministry of Education, College of Chemical and Biological Engineering, Zhejiang University, Hangzhou 310027, China; Research Center of Industrial Ecology, Institute of Zhejiang University – Quzhou, Quzhou 324000, China; Key Laboratory of Biomass Chemical Engineering of Ministry of Education, College of Chemical and Biological Engineering, Zhejiang University, Hangzhou 310027, China; Research Center of Industrial Ecology, Institute of Zhejiang University – Quzhou, Quzhou 324000, China; Key Laboratory of Biomass Chemical Engineering of Ministry of Education, College of Chemical and Biological Engineering, Zhejiang University, Hangzhou 310027, China; Key Laboratory of Biomass Chemical Engineering of Ministry of Education, College of Chemical and Biological Engineering, Zhejiang University, Hangzhou 310027, China; State Key Laboratory of Advanced Technology for Materials Synthesis and Processing, Wuhan University of Technology, Wuhan 430070, China; Key Laboratory of Biomass Chemical Engineering of Ministry of Education, College of Chemical and Biological Engineering, Zhejiang University, Hangzhou 310027, China; Shanghai Synchrotron Radiation Facility, Shanghai Advanced Research Institute, Chinese Academy of Sciences, Shanghai 201800, China; Institut de Science et d'Ingénierie Supramoléculaires, University of Strasbourg, Strasbourg F-67000, France; Key Laboratory of Biomass Chemical Engineering of Ministry of Education, College of Chemical and Biological Engineering, Zhejiang University, Hangzhou 310027, China; Hydrogen Energy Institute, Zhejiang University, Hangzhou 310027, China; School of Biological and Chemical Engineering, NingboTech University, Ningbo 315100, China

**Keywords:** CO_2_ electroreduction, acidic electrolyte, methane, covalent hydrophobic interface, single-pass carbon efficiency

## Abstract

Electrochemical conversion of CO_2_ to methane provides a sustainable pathway for fuel synthesis, yet it inherently struggles to balance carbon utilization efficiency with product selectivity. Conventional surface engineering based on physical hydrophobic coatings often leads to interfacial instability and diminished charge transfer efficiency. To address these issues, we develop a cysteine-coated copper coordination complex catalyst modified with covalently bonded fluoroalkyl silane (FAS), allowing precise control over surface wettability. A breakthrough in highly acidic electrolytes is demonstrated, achieving a methane Faradaic efficiency of up to 66.2% at 400 mA cm^−2^ (pH 1.8), alongside a single-pass carbon conversion efficiency of 31.1%, surpassing conventional alkaline-system benchmarks. Surface-enhanced Raman spectroscopy reveals a key *COOH intermediate for CO_2_ activation, while *in situ* attenuated total reflectance Fourier transform infrared (ATR-FTIR) spectroscopy monitors the sequential hydrogenation pathway through *CHO and *CH_2_O. Molecular dynamics simulations further reveal a distinct water exclusion zone near the catalyst surface, which arises from the hydrophobic covalent interface induced by the FAS coating. This interfacial engineering strategy suppresses the hydrogen evolution reaction by blocking water access, preserves hydrophobicity during operation, and offers a scalable path to improve the kinetics and selectivity of CO_2_ electroreduction.

## Teaser

This study develops a molecularly engineered covalent hydrophobic interface that enables highly efficient electrocatalytic reduction of CO2 to methane under strongly acidic conditions, achieving a record-high single-pass carbon efficiency for sustainable fuel production."

## INTRODUCTION

Electrochemical CO_2_ reduction (CO_2_ERR) powered by renewable electricity presents a carbon-neutral pathway for converting CO_2_ and water into value-added fuels under ambient conditions [1–4], with the product methane (CH_4_) emerging as a strategic energy carrier due to its high energy density and infrastructure compatibility [5,6]. Current challenges primarily arise from competing reaction pathways and intrinsic kinetic barriers associated with the multi-step deep reduction process required for CH_4_ production [7–12]. Molecular catalysts effectively mitigate these challenges by virtue of their atomically precise active sites, which not only enhance charge transfer efficiency but also effectively suppress competitive side reactions, such as hydrogen evolution [13–17], enabling selective hydrocarbon production. This combination of structural precision and dynamic control makes molecular catalysts essential components for efficient CO_2_ERR systems.

Despite the prevalent use of alkaline or neutral electrolytes, current CO_2_ERR systems are still plagued by inherent (bi)carbonate issues [18,19]. The interaction between CO_2_ and hydroxide induces non-Faradaic carbonate formation, resulting in substantial CO_2_ loss and limiting carbon utilization efficiency to under 20% [20]. Furthermore, bicarbonate deposition blocks gas transport, compromises CO_2_ diffusion, and accelerates reactor degradation. Therefore, CO_2_ERR inherently avoids carbonate formation in acidic media. Protons from the electrolyte suppress hydroxide accumulation, while any carbonates dissolve rapidly under acidic conditions [21–23]. However, high proton concentrations also promote competing hydrogen evolution. Thus, achieving stable and selective conversion of CO_2_ to CH_4_ in an acidic electrolyte remains a significant challenge for industrial carbon utilization. Beyond the dominant hydrogen evolution reaction, key hurdles include the kinetic sluggishness of the multi-step proton-coupled electron transfers required for CH_4_ formation, and the susceptibility of catalyst structures to corrosion or reconstruction under harsh acidic conditions. To address these challenges, researchers have developed several interfacial engineering strategies [24–27]. These include modulating the local cation environment to repel competing protons, designing molecular catalysts for precise intermediate binding, and employing hydrophobic layers to enrich CO_2_ at the electrode–electrolyte interface. Through precisely tailored surface wettability, it is possible to construct stable triple-phase reaction boundaries that restrict excessive proton flux while simultaneously enhancing the local CO_2_ concentration at catalytic sites.

Recent advances in interface engineering have opened new pathways to tackle the core challenges of acidic CO_2_ERR. These innovative approaches focus on precisely tailoring the electrode–electrolyte interface to establish favorable microenvironments that suppress hydrogen evolution while promoting CO_2_ conversion. Molecular layer engineering offers a powerful route through immobilizing nitrogen-containing imidazole layers, allowing dynamic regulation of the hydrogen source according to the applied potential, achieving high formate efficiency by switching between proton transfer and hydrogen bond disruption mechanisms [28]. Additionally, crown ether modification of copper catalysts, such as with 18-Crown-6, promotes acidic CO_2_ to CH_4_ conversion by specifically coordinating potassium ions to create a distinct interfacial setting. This enhances the local electric field to stabilize the *CO and facilitates protonation via hydrolysis, leading to a notable 51.2% CH_4_ yield [20]. Furthermore, elaborately designed single-molecule heterojunction catalysts attain near-perfect CO selectivity and sustain stable operation for over 200 h at pH 1 [29]. Together, these interfacial engineering strategies illustrate that carefully modulating the local chemical environment, through electric field enhancement, intermediate stabilization and proton transport control, provides a versatile and effective means to overcome the inherent limitations of acidic CO_2_ERR.

Conventional engineering strategies that rely on physically coated hydrophobic layers, e.g. polytetrafluoroethylene (PTFE), suffer from interfacial instability during high-current-density CO_2_ERR operation [30]. This instability not only compromises long-term durability but also leads to electrode flooding and disruption of charge transfer. In contrast, the covalent integration of hydrophobic motifs formed via molecular self-assembly enables construction of chemically stable and well-anchored architectures [31–33]. This strategy synergistically enhances interfacial bonding stability while allowing precise control of hydrophobicity, thereby maintaining efficient charge transfer. Furthermore, it enables the tailored manipulation of the local chemical environments, such as interfacial structure, proton gradients and reactant concentration, to optimize the kinetics and thermodynamics of CO_2_ reduction [34–36]. Such a covalently engineered hydrophobic interface is anticipated to deliver record performance by improving mass transport and regulating water distribution to promote deep hydrogenation, boosting the CO_2_-to-CH_4_ conversion while maintaining high activity, selectivity and operational stability in CO_2_ electrolysis.

**Figure 1.**
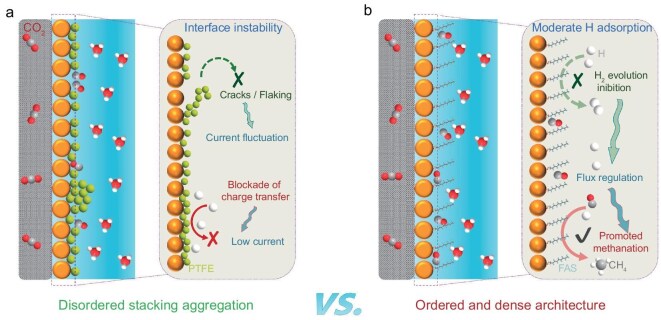
Schematic illustration of CO_2_ERR in acidic electrolytes. (a) Physically coated PTFE hydrophobic electrode. (b) Covalently bonded FAS hydrophobic electrode for CO_2_ methanation in strongly acidic media.

Herein, we report a covalently engineered cysteine-anchored copper complex integrated with a hydrophobic interface via *in situ* self-assembly of fluoroalkyl silane (FAS). This molecular-level covalent integration creates a proton-selective interface with finely regulated hydrophobicity, effectively addressing the interfacial instability and charge transfer constraints associated with conventional physical coatings. The resulting electrode enables CO_2_ERR-to-CH_4_ conversion with a Faradaic efficiency (FE) of 66.2%, accompanied by a partial current density of 264.7 mA cm^−2^, and notably achieves a single-pass carbon efficiency (SPCE) of 31.1% in a strongly acidic medium at pH 1.8. The covalently bonded FAS hydrophobic interface, constructed via molecular self-assembly, successfully circumvents electrode flooding and charge transfer disruption caused by insufficient interfacial adhesion in physically coated PTFE layers under high current densities (Fig. 1). Molecular dynamics (MD) simulations confirm this superiority, radial distribution function analysis reveals that the FAS excludes water molecules beyond 15 Å, while pristine copper exhibits unrestricted water accumulation. By leveraging optimized hydrophobicity that synergistically balances gas diffusion, proton supply and electron transport kinetics, this interfacial engineering delivers both high selectivity and operational stability.

## RESULTS AND DISCUSSION

**Figure 2.**
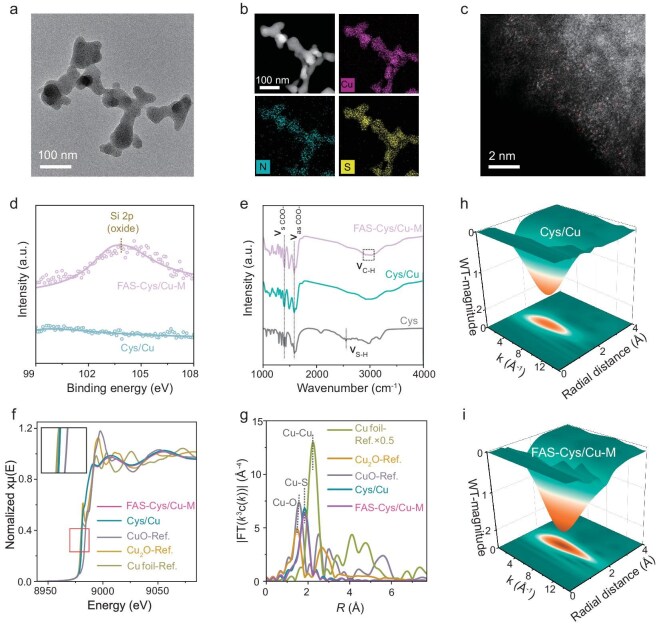
Structural characterizations of Cys/Cu and FAS–Cys/Cu-M. (a) TEM image. (b) EDX elemental mapping images. (c) AC HAADF-STEM image of Cys/Cu. (d) High-resolution Si 2p XPS spectra of Cys/Cu and FAS–Cys/Cu-M. (e) FT-IR spectra of Cys/Cu, FAS–Cys/Cu-M and Cys ligands. (f) Cu K-edge XANES and (g) FT-EXAFS spectra for Cys/Cu, FAS–Cys/Cu-M and reference samples. Inset: the corresponding magnified pre-edge XANES region. WT for the *k*^3^-weighted Cu K-edge EXAFS signals of (h) Cys/Cu and (i) FAS–Cys/Cu-M.

We developed a molecularly engineered electrode through a facile one-step self-assembly of cysteine-coated copper coordination complexes (Cys/Cu), functionalized with an FAS molecular layer via silanization to construct a robust hydrophobic layer. Through precisely controlled covalent functionalization with FAS, by tuning the degree of FAS coverage in the chemisorbed self-assembled monolayer, we engineered a series of Cys/Cu electrodes with tunable hydrophobicity, categorized into three distinct tiers: FAS–Cys/Cu-L (low hydrophobicity), FAS–Cys/Cu-M (medium hydrophobicity) and FAS–Cys/Cu-H (high hydrophobicity), enabling systematic investigation of interfacial wettability effects on performance. Transmission electron microscopy (TEM) of Cys/Cu shows no visible metallic nanoparticles or aggregates (Fig. 2a). Fast Fourier transform (FFT) patterns further reveal the absence of metallic diffraction signatures (Fig. S1), supporting the lack of distinct Cu phases. Energy-dispersive X-ray spectroscopy (EDX) elemental mapping demonstrates the uniform distribution of Cu throughout the framework, with their elemental signals overlapping well with those of S and N (Fig. 2b). The atomic dispersion of Cu is directly evidenced by aberration-corrected high-angle annular dark-field scanning transmission electron microscopy (AC HAADF-STEM) imaging, where uniformly dispersed, isolated bright dots correspond to Cu species, with no detectable metallic Cu particles or clusters (Fig. 2c).

We focused first on FAS–Cys/Cu-M as a representative system, with the other two samples discussed subsequently for the sake of comparison. Scanning electron microscopy (SEM) imaging of the pristine Cys/Cu reveals its surface morphology, characterized by distinct agglomerates and significant roughness. In contrast, the FAS-functionalized FAS–Cys/Cu-M retains the original morphology (Fig. S2). SEM-EDX mapping images display uniform elemental distribution (Figs S3 and S4), indicating that FAS deposition preserves the structural and chemical integrity of the Cys/Cu framework. X-ray diffraction (XRD) analysis shows no metallic Cu or oxide phases in either FAS–Cys/Cu-M or Cys/Cu samples (Fig. S5). X-ray photoelectron spectroscopy (XPS) analysis reveals no distinct satellite peak around 943 eV in either material, which is consistent with a minimal presence of Cu^2+^ (Fig. S6). Detected F 1s signals correspond to those of FAS and Nafion (Fig. S7). Critically, the presence of Si 2p signals on the surface of FAS–Cys/Cu-M confirms successful FAS deposition (Fig. 2d), which is supported by EDX mapping (Fig. S8). Collectively, these results demonstrate the successful synthesis of FAS-modified Cys/Cu catalysts with well-dispersed Cu sites.

Fourier-transform infrared (FT-IR) spectra show structural evolution from cysteine ligands to Cys/Cu and FAS–Cys/Cu-M samples (Fig. 2e). The appearance of –COO^−^ vibrations at 1583 cm^−1^ (asymmetric) and 1402 cm^−1^ (symmetric), along with the disappearance of the –S–H stretch at 2530 cm^−1^ in Cys/Cu, confirm successful S–Cu coordination [6,37]. In FAS–Cys/Cu-M, the emergence of –C–H stretches around 2931 cm^−1^ corroborates FAS deposition [33]. This observation is in line with established mechanisms [31,33], whereby FAS hydrolysis produces silanols that covalently graft onto the hydroxylated Cys/Cu surface via dehydration (Fig. S9). This results in an aligned perfluoroalkyl siloxane network that combines superhydrophobicity with electrochemical activity. We further elucidated the electronic configuration and coordination environment of Cu atoms using Cu K-edge X-ray absorption spectroscopy. The X-ray absorption near-edge structure (XANES) spectra show that the near-edge absorption energies of FAS–Cys/Cu-M and Cys/Cu are very close and located between Cu_2_O and CuO, and closer to Cu_2_O (Fig 2f, Fig. S10). The coordination structure of Cu atoms was studied by *k*^3^-weighted Fourier-transformed extended X-ray absorption fine structure (FT-EXAFS) spectra. Both FAS–Cys/Cu-M and Cys/Cu show a similar main peak at ∼1.8 Å in *R*-space (Fig. 2g), corresponding to the Cu–S scattering path. No metallic Cu–Cu signals at ∼2.2 Å can be observed for FAS–Cys/Cu-M or Cys/Cu, confirming the existence of atomic Cu sites, which agrees well with the AC HAADF-STEM images. These results provide evidence that Cu is present in a cationic state stabilized by cysteine coordination. Fitting of the FT-EXAFS data further reveals a Cu–S coordination number of 2.5 ± 0.2 in FAS–Cys/Cu-M (Fig. S11, Table S1). Moreover, the visualized coordination configuration in wavelet-transform (WT) analysis of EXAFS in both *R*- and *k*-spaces further confirms the predominant Cu–S path. After covalent grafting of FAS, the Cys/Cu system still maintains its single-atom dispersion state without compromising the monodispersity of Cu sites (Fig. 2h). Analysis of Cu-specific WT contour plots reveals negative shifts in both bond length (R) and wavevector (K) parameters for Cys/Cu and FAS–Cys/Cu-M compared to Cu foil (Fig. 2i, Fig. S12), confirming the exclusion of metallic Cu crystallites within the catalytic system.

**Figure 3.**
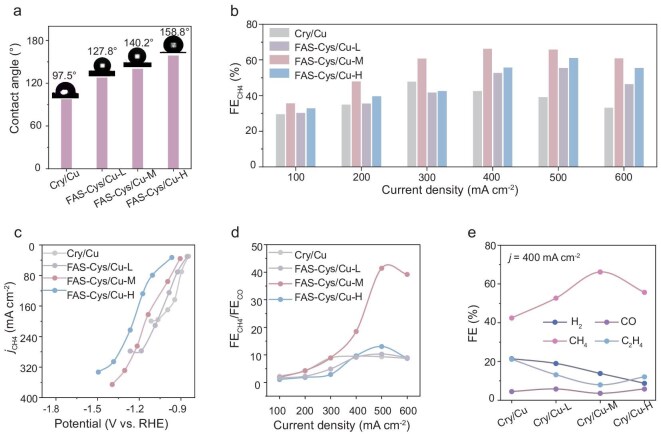
Effect of electrode wettability on CO_2_ electromethanation. (a) Water contact angles for Cys/Cu and FAS-modified Cys/Cu. (b) FE_CH4_ on a series of catalysts at different current densities. (c) CH_4_ partial current densities under various applied potentials, and (d) ratios of FE_CH4_ to FE_CO_ at different current densities for Cys/Cu, FAS–Cys/Cu-L, FAS–Cys/Cu-M and FAS–Cys/Cu-H. (e) Comparison of the FE of H_2_, CO, CH_4_ and C_2_H_4_ at 400 mA cm^−2^.

Water contact angle measurements show that increased FAS loading amounts enhance surface hydrophobicity; pristine Cys/Cu is weakly hydrophobic (97.5°), while FAS–Cys/Cu-H becomes superhydrophobic (158.8°). FAS–Cys/Cu-M and FAS–Cys/Cu-L exhibit intermediate contact angles of 140.2° and 127.8°, respectively (Fig. 3a, Fig. S13). We evaluated the CO_2_ERR performances in a flow cell using a gas diffusion electrode loaded with the catalyst (Fig. S14). In a strongly acidic electrolyte (3.0 M KCl + H_2_SO_4_, pH 1.8) (Table S2), CH_4_ was identified as the main product for all investigated catalysts. FAS–Cys/Cu-M showed optimal CH_4_ selectivity, with FEs exceeding 60% in the range of 300–600 mA cm^−2^ and reaching a maximum of 66.2% at 400 mA cm^−2^ (Fig. S15). A volcano-shaped correlation was observed between CH_4_ FE and electrode wettability modulated by FAS loading (Fig. 3b). The weakly hydrophobic Cys/Cu achieved only a moderate CH_4_ FE of 47.8% at 300 mA cm^−2^. Excessive hydrophobicity proved detrimental, as evidenced by the reduced CH_4_ FE (61.1%) of FAS–Cys/Cu-H. These findings demonstrate that moderate hydrophobicity optimally balances interfacial water, promoting proton accessibility while minimizing excessive water flooding, and enhancing CH_4_ selectivity [38]. This reduction in performance under high hydrophobicity can be attributed to mass transport limitations, where the overly thick FAS layer restricts proton access and CO_2_ diffusion to the active sites, thereby impeding key hydrogenation steps and reducing CH_4_ selectivity.

Further, the FAS–Cys/Cu-M achieved a high CH_4_ partial current density of 264.7 mA cm^−2^ at a low potential of −1.21 V, with a further increase to 365.4 mA cm^−2^ observed at −1.31 V (Fig. 3c). This indicates an efficient CH_4_ production pathway at reduced overpotentials. Notably, FAS–Cys/Cu-M achieved a CH_4_/CO ratio of 41.5 at 500 mA cm^−2^, which is 4.0 and 3.2 times higher than those of FAS–Cys/Cu-L (10.3) and FAS–Cys/Cu-H (13.0), respectively (Fig. 3d), underscoring the importance of optimized wettability in product selectivity. In addition, FAS–Cys/Cu-M exhibited the highest CH_4_/C_2_H_4_ and CH_4_/(CO + C_2_H_4_) ratios among all tested samples (Fig. S16), demonstrating its overall superiority toward the CH_4_ pathway. Although FAS modification introduces a kinetic overpotential penalty at high current densities (Fig. S17), it establishes a hydrophobic microenvironment that effectively suppresses the hydrogen evolution reaction. This is also confirmed by the lower H_2_ FE of FAS–Cys/Cu-M (13.8%) compared to Cys/Cu (21.4%) at 400 mA cm^−2^ (Fig 3e, Fig. S18). Electrochemical analysis revealed a lower double-layer capacitance for FAS–Cys/Cu-M (2.52 mF cm^−2^) than Cys/Cu (3.91 mF cm^−2^) (Fig. S19), indicating a reduced electrode–electrolyte interface. Despite this diminished electrical contact, however, the tailored hydrophobicity enhances CO_2_ diffusion through an optimized triple-phase boundary [24], leading to the superior activity.

**Figure 4.**
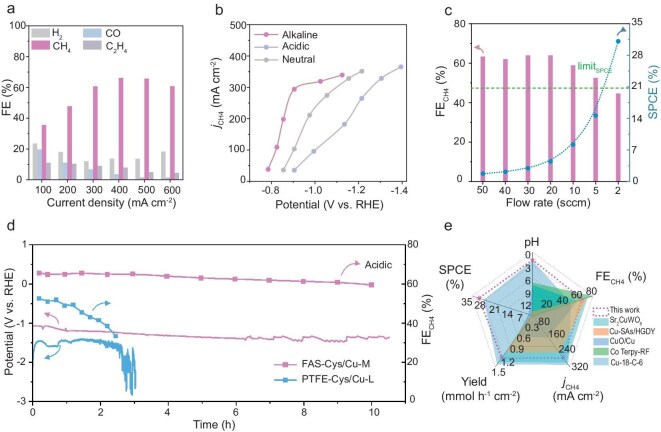
Performance for CO_2_ electromethanation. (a) Gas product FEs of CO_2_ERR over FAS–Cys/Cu-M in acidic electrolyte. (b) The partial current density of CH_4_ at different potentials in alkaline electrolyte, neutral electrolyte and acidic electrolyte. (c) FE and SPCE of CO_2_-to-CH_4_ in an acidic electrolyte with different CO_2_ flow rates. (d) Long-term stability of CO_2_ERR in an acidic electrolyte. (e) Comparison of this work with state-of-the-art catalysts, including electrolyte pH, FE_CH4_, *j*_CH4_, CH_4_ yield and SPCE.

We initially assessed the CO_2_ERR performance of the optimal FAS–Cys/Cu-M over a wide pH range to clarify the influence of electrolyte acidity (Fig. S20). Although a peak CH_4_ FE of 73.5% was achieved at 400 mA cm^−2^ in alkaline electrolyte (1.0 M KOH, pH 13.5) and 70.4% at 300 mA cm^−2^ under neutral conditions (1.0 M KHCO_3_, pH 8.3), in both cases, the high performance was followed by a sharp decrease attributable to electrode flooding and carbonate deposition. Notably, acidic media enabled sustained high selectivity, maintaining over 60% CH_4_ FE across 300–600 mA cm^−2^ with a 66.2% maximum at 400 mA cm^−2^ (Fig. 4a). The higher working potential observed in acidic media relative to alkaline/neutral systems (Fig. 4b) mainly stems from inherent differences in the charge transfer kinetics of the electrolyte. Together, the results establish acidic conditions as optimal for efficient CO_2_-to-CH_4_ conversion.

We further evaluated the CO_2_ utilization efficiency via SPCE under varying CO_2_ flow rates. FAS–Cys/Cu-M achieved a significant SPCE of 31.1% at 2 mL min^−1^ (Fig. 4c), surpassing the theoretical 20% limit typically observed in neutral or alkaline systems [20]. The SPCE value of 31.1% is notable, as it represents the proportion of inlet CO_2_ converted into the target product CH_4_, demonstrating a substantial improvement in carbon utilization efficiency. Our acidic system overcomes the issue of salt deposition and achieves more effective CO_2_ conversion. In acidic electrolyte, the electrolysis system also exhibited high product selectivity, with CH_4_ accounting for 90.1% of all CO_2_ERR gas products at 500 mA cm^−2^ (Fig. S21). Durability tests revealed pH-dependent stability; while performance remained stable for over 10 h in acidic media with negligible selectivity loss (Fig. 4d), gradual degradation occurred after 6 h in alkaline conditions (Fig. S22). Notably, the acidic system exhibited exceptional pH stability, with a pH fluctuation of only 0.36 observed throughout prolonged electrolysis (Table S3). Post-reaction imaging revealed substantial salt deposition on the alkaline electrolyte, in contrast to minimal deposition under acidic conditions (Fig. S23). The characterization of the post-electrolysis samples confirmed the structural and chemical stability of the catalyst under operating conditions. SEM images revealed the structural integrity of the catalyst before and after the reaction (Fig. S24), while XRD patterns showed no detectable diffraction peaks corresponding to metallic Cu or oxides (Fig. S25), ruling out atomic agglomeration during prolonged operation. XPS analysis indicated no obvious changes in oxidation states (Fig. S26). Post-reaction Cu K-edge XANES spectra showed minimal deviation from the pre-reaction data, indicating stability of the Cu species’ electronic structure. This is supported by the Fourier-transformed EXAFS spectra, which display a stable Cu–S coordination peak near 1.8 Å and an absence of Cu–Cu scattering paths (Fig. S27). Inductively coupled plasma optical emission spectrometry (ICP-OES) analysis confirmed that the concentration of Cu in the post-reaction acidic electrolyte was below the instrument’s detection limit, indicating no significant Cu dissolution occurred during the stability test (Table S4). This distinctive interfacial structure endows FAS–Cys/Cu-M with superior performance compared to state-of-the-art CO_2_-to-CH_4_ catalysts across multiple key metrics, including operating electrolyte pH, CH_4_ FE, CH_4_ partial current density (*j*_CH4_), CH_4_ yield and SPCE (Fig. 4e, Tables S5 and S6).

**Figure 5.**
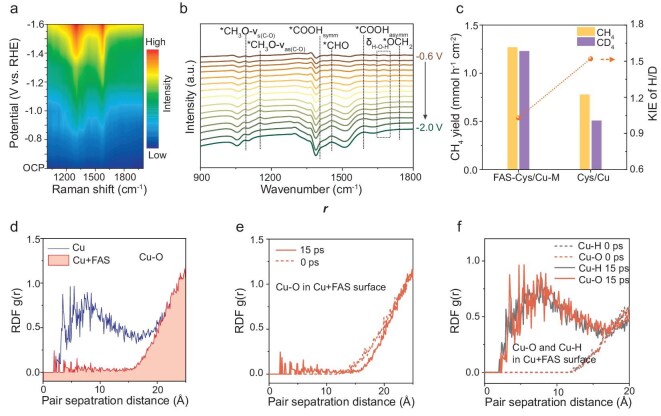
Mechanism analysis. (a) *Operando* surface-enhanced Raman spectra and (b) *in situ* ATR-FTIR spectra of FAS–Cys/Cu-M during the CO_2_ERR. (c) CH_4_ yield and KIE of H/D substitution of FAS–Cys/Cu-M and Cys/Cu in acidic media at 400 mA cm^−2^. (d) The RDF of Cu–O in Cu and Cu–FAS surfaces at 15 ps. (e) The RDF of Cu–O in Cu–FAS at 0 and 15 ps. (f) The RDF of Cu–O and Cu–H in Cu at 0 and 15 ps.

*Operando* surface-enhanced Raman spectroscopy was used to probe the reaction pathway and real-time interfacial changes during the CO_2_ERR process (Fig. 5a). Distinct vibrational bands at 1321 and 1578 cm^−1^ were observed, corresponding to the symmetric [ν_s(O–C–O)_] and asymmetric [ν_as(O–C–O)_] stretching modes of adsorbed *COOH, respectively [39]. These signals indicate the effective adsorption and activation of the CO_2_ molecule [40]. *In situ* attenuated total reflectance Fourier transform infrared (ATR-FTIR) spectroscopy further captured key intermediates along the reaction pathway (Fig. 5b). The *COOH intermediate was detected at 1410 cm^−1^ (symmetric) and 1585 cm^−1^ (asymmetric) [41], respectively, with its dynamic evolution governing CH_4_ selectivity. The detection of subsequent hydrogenation steps, as evidenced by the emergence of *CHO (1454 cm^−1^) and *CH_2_O (1740 cm^−1^) [42,43], suggests a sequential proton-coupled electron transfer process. The gradual weakening of C–O bonds during *CO hydrogenation was reflected in the symmetric (1086 cm^−1^) and asymmetric (1149 cm^−1^) stretching vibrations of *CH_3_O [44,45], directly correlating bond activation with CH_4_ formation efficiency. Together, these results provide definitive spectroscopic validation of the stepwise hydrogenation mechanism leading to CH_4_. The sequential detection of *COOH, *CHO and *CH_2_O identified in our spectroscopic analysis aligns directly with the established CO_2_-to-CH_4_ hydrogenation mechanism; *COOH formation initiates CO_2_ activation, *CHO generation represents the critical C–H bond formation step, and *CH_2_O emergence corroborates deep hydrogenation toward CH_4_ (Fig. S28).

We further conducted kinetic isotope effect (KIE) studies using H/D substitution on both Cys/Cu and FAS–Cys/Cu-M, confirming enhanced proton-transfer kinetics at the modulated interfacial environment. The FAS–Cys/Cu-M exhibits a KIE value of 1.03 (Fig. 5c), suggesting negligible isotope dependence and thus indicating that proton transfer is no longer the rate-limiting step. This stands in sharp contrast to the KIE of 1.52 observed for Cys/Cu, where proton transfer dominates the reaction kinetics [44]. The reduction in KIE provides direct evidence that covalent interfacial modification decouples the hydrogenation process from proton-transfer limitations. Moreover, the lower KIE under acidic conditions (1.03) compared to alkaline media (1.17) underscores the role of electrolyte proton availability in facilitating efficient protonation (Figs S29 and S30), whereas the higher KIE in alkaline conditions highlights the kinetic barrier associated with water dissociation [46].

We conducted MD simulations to probe the critical role of FAS in modulating the electrode–electrolyte interface by comparing water behavior near pristine and FAS-functionalized surfaces. Radial distribution function (RDF) analysis of Cu–O and Cu–H pairs reveals distinct water behaviors at the two interfaces. On the FAS-modified surface, sustained oscillations in the Cu–O RDF below 15 Å reflect the confined spatial distribution of a single adsorbed water molecule. Pronounced peaks beyond 15 Å indicate effective exclusion of water from the hydrophobic FAS layer (Fig. 5d). In contrast, the unmodified surface exhibits continuous Cu–O peaks starting from interatomic distances (>2 Å), indicating unrestricted water diffusion and adsorption. Time-dependent RDF comparisons (0 vs. 15 ps) further demonstrate the blocking effect of the FAS layer (Fig. 5e); weak oscillations below 12 Å at 15 ps originate from dynamic fluctuations of the residual adsorbed water (Fig. 5f). On the unmodified surface, the absence of Cu–O signals within 12 Å at 0 ps and the emergence of intense oscillatory features at 15 ps signify progressive water accumulation and the formation of a structured interfacial water layer, consistent with previously reported behavior [47,48]. The RDF analysis of Cu–H bonds reveals that the spatial distribution variation of the hydrogen ends of water molecules from the initial moment (0 ps) to equilibrium (15 ps) remains highly consistent with the Cu–O RDF. Over time, the conformation of water gradually approaches the Cu surface, forming a hydration-like structural feature near the surface region. This process, together with the synergistic changes in the Cu–O bond RDF, collectively reveals the dynamic reconstruction of the interfacial water network.

**Figure 6.**
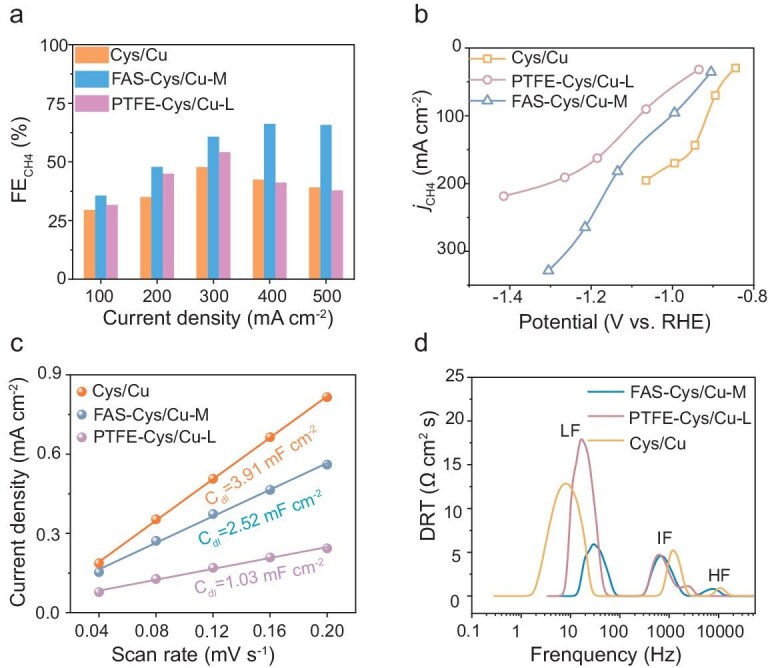
Comparing chemically bonded with physically coated hydrophobic layers in CO_2_ERR. (a) FE and (b) partial current densities of CH_4_ for Cys/Cu, PTFE–Cys/Cu-L and FAS–Cys/Cu-M. (c) Electrochemical active surface area (ECSA) and (d) DRT analysis for Cys/Cu, PTFE–Cys/Cu-L and FAS–Cys/Cu-M.

To evaluate the advantages of the covalently engineered interface, comparative experiments were conducted using Cys/Cu modified with physically deposited PTFE (Fig. 6a). At 300 mA cm^−2^, the low-loading PTFE–Cys/Cu-L delivered a CH_4_ FE of 54.1%, while the high-loading PTFE–Cys/Cu-H reached only 41.2% under the same conditions (Fig. S31), both significantly lower than that of the covalently modified FAS–Cys/Cu-M. This performance trend was further reflected in the CH_4_ partial current densities, where FAS–Cys/Cu-M substantially outperformed both PTFE–Cys/Cu-L and the unmodified Cys/Cu (Fig. 6b). Although PTFE–Cys/Cu-L showed improved CH_4_ selectivity and a higher CH_4_/C_2_H_4_ ratio compared to the bare electrode (Fig. S32), its performance still lagged behind that of FAS–Cys/Cu-M. The inferior behavior of the high-loading PTFE is ascribed to excessive polymer coverage, which restricts mass transport and compromises charge transfer efficiency [49]. These results collectively affirm that covalent interfacial engineering provides a more robust and efficient pathway for sustaining high CO_2_-to-CH_4_ conversion under demanding operational conditions. This further underscores that high hydrophobicity in physical coatings like PTFE exacerbates mass transport limitations by forming unstable barriers that block reactant access, leading to flooding and charge transfer inefficiency, which contrasts with the durable covalent interface of FAS.

Furthermore, the PTFE–Cys/Cu-L delivered poor performance, characterized by low CH_4_ FE and unstable operating potentials (Fig. 4d), which can be attributed to degradation of its physically deposited hydrophobic layer. This instability was corroborated by a marked decrease in water contact angle from 113.5° to 86.3° after electrolysis (Figs S33 and S34), indicating progressive surface wetting, consistent with MD predictions for unmodified Cu surfaces. Electrochemical analyses further revealed inferior interfacial characteristics of PTFE–Cys/Cu-L. The double-layer capacitance (*C*_dl_) was only 1.03 mF cm^−2^, lower than the 2.52 mF cm^−2^ measured for FAS–Cys/Cu-M (Fig. 6c, Fig. S35), indicating a reduced electroactive area. Concurrently, impedance spectroscopy showed higher charge transfer resistance (Fig. S36), collectively pointing to inefficient electron transfer at the electrode–electrolyte interface. Distribution of relaxation times (DRT) analysis further identified prominent low-frequency peaks for PTFE–Cys/Cu-L, suggestive of mass transport limitations (Fig. 6d). In contrast, FAS–Cys/Cu-M exhibited suppressed low-frequency features and a shift toward higher-frequency processes, reflecting enhanced kinetics. Although Cys/Cu displayed intermediate-frequency peaks associated with proton transfer constraints, FAS–Cys/Cu-M maintained stable hydrophobicity throughout operation, underscoring the essential role of covalent stabilization in achieving durable CO_2_ERR performance.

## CONCLUSIONS

In summary, this study demonstrates an efficient CO_2_-to-CH_4_ conversion system operable in strongly acidic media through covalent hydrophobic engineering of a Cys/Cu using FAS. The chemically grafted FAS layer constructs a stable triple-phase interface that precisely tailors surface wettability and proton supply, enabling a CH_4_ FE of 66.2% and an SPCE of 31.1% at 400 mA cm^−2^ in acidic electrolyte (pH 1.8). The electrode also exhibits remarkable pH universality, achieving CH_4_ FE exceeding 73.5% in alkaline media and over 70% in neutral media. In contrast to physically coated PTFE, which suffered from progressive wettability degradation, the covalently anchored FAS maintained robust hydrophobicity throughout operation, effectively preventing electrode flooding and salt deposition. MD simulations corroborate that the FAS interface effectively excludes water over an extended region, thereby suppressing hydrogen evolution and facilitating CO_2_ diffusion. This work provides a scalable interfacial design strategy that concurrently enhances reaction kinetics and product selectivity in CO_2_ERR.

## METHODS

### Catalyst synthesis

Cys/Cu was synthesized in a one-step self-assembly process in aqueous solution. Specifically, 600 mg of CuSO_4_·5H_2_O was dissolved in 50 mL of ultrapure water under sonication for 5 min, followed by the addition of 1.6 g of cysteine (Cys) and stirring for 10 min. After aging for 30 min, the product was collected by centrifugation (9000 r/min, 5 min) and washed with water and ethanol three times. The final product was freeze-dried and stored at low temperatures.

To fabricate the cathode loaded with Cys/Cu, 10 mg of Cys/Cu powder was dispersed in 1 mL of *n*-propanol containing 20 μL of Nafion perfluorinated resin solution (5 wt.%) via ultrasonication. The resulting catalyst slurry was slowly drop-cast onto a hydrophobized carbon paper to achieve a loading of ∼1.0 mg cm^−2^, and dried completely before use. The electrode was then modified with FAS using a liquid self-growth method to tune surface hydrophobicity. Specifically, solutions of methanol and FAS at volume ratios of 50:1, 100:1 and 200:1 were prepared, and the Cys/Cu was immersed at 70°C for 1 h to introduce hydrophobic functional groups. Finally, the hydrophobic electrodes were vacuum-dried and categorized as having high, moderate and low hydrophobicity, designated as FAS–Cys/Cu-H, FAS–Cys/Cu-M and FAS–Cys/Cu-L, respectively. For comparison, PTFE coatings were applied onto pre-prepared Cys/Cu at loadings of 5 and 15 wt.%, followed by drying at room temperature. These samples were designated as PTFE–Cys/Cu-L and PTFE–Cys/Cu-H, respectively.

### Electrochemical measurements

The performance of CO_2_ERR over as-prepared catalysts was performed in a flow cell equipped with a three-electrode system on the electrochemical workstation (CHI 760E). For performance studies, 1.0 M KOH, 1.0 M KHCO_3_ or 3.0 M KCl with H_2_SO_4_ was used as the electrolyte. The catholyte was continuously circulated at a rate of 20 sccm. Both the anodic and cathodic electrolyte chambers in the flow cell were separated by the anion/proton exchange membrane. High-purity CO_2_ gas (99.999%) was injected into the chamber behind the working electrode at a flow rate of 20 mL min^−1^. The gas products from the cathodic chamber were vented directly into the gas sampling loop of the gas chromatograph. The working area of the electrode was set to be 1.0 cm^2^. The reference electrode was an Ag/AgCl electrode, and the counter electrode was an Ni foam. All the potentials were recorded against an Ag/AgCl (saturated KCl) reference electrode and then converted to the reversible hydrogen electrode (RHE) scale using the equation: *E* (vs. RHE) = *E* (vs. Ag/AgCl) + 0.197 V + 0.0591 V × pH + *iR*. The resistance *R* determined by impedance spectroscopy was used to correct the measured potentials in the polarization curves via post-measurement correction. To mitigate the risk of overcompensation, 85% of the measured *R* was applied during this *iR* compensation step. All potentials applied in the main text and the Supplementary data refer to RHE unless otherwise stated.

### *In situ* Raman and *in situ* ATR-FTIR characterizations

*In situ* shell-isolated nanoparticle-enhanced Raman spectroscopy measurements were carried out using an XploRA PLUS Raman spectrometer (HORIBA Jobin Yvon). A magnification of 50× with a long-distance 8 mm objective was used. The excitation wavelength was 638 nm from a He–Ne laser. The Raman spectrometer was calibrated using an Si wafer. A custom-made spectroelectrochemical cell with a Pt wire counter electrode and an Ag/AgCl reference electrode was used. The BRUKER INVENIO R spectrometer, equipped with a liquid nitrogen-cooled mercury cadmium telluride (MCT) detector, was utilized for *in situ* ATR-FTIR measurements. An Ag/AgCl electrode served as the reference electrode, while a Pt foil was employed as the counter electrode. During the collection of spectra, the optical path was continuously purged with CO_2_ gas to minimize interference from water vapor. The background spectrum of the catalyst electrode was recorded at an open-circuit potential prior to each measurement. The absorbance spectra [−log(*R*/*R*_0_)] at different potentials were then collected at a spectral resolution of 8 cm^−1^.

### Theoretical calculations

The MD simulation was implemented by CP2K [50]. The Goedecker–Teter–Hutter pseudopotentials were used to describe the valence and core electrons of the system. The Gaussian basis set was double-ς with one set of polarization functions. The plane wave cutoff was set at 350 Ry. The Perdew–Burke–Ernzerhof functional was utilized for the exchange-correlation effects, and the Grimme D3 method was considered in dispersion correction. The canonical ensemble was imposed with Nosé–Hoover thermostat treatment (298 K). The MD was performed by 15 ps with a time step of 1 fs. For the Cu model, a three-layer Cu (111) basement (108 Cu atoms and 84 H_2_O) was built with lattice parameters of *a* = *b* = 15.336 Å, *c* = 47.619 Å. The four FAS were anchored to the Cu surface through Cu–O bonds. Because of the large system of Cu and Cu-FAS models, only the gamma point was used in all calculations.

## Supplementary Material

nwag116_Supplemental_File
