# Supplementary material for: Molecularly engineered covalent hydrophobic interface for enhanced CO2 electromethanation in strong acid
Source: Natl Sci Rev. 2026 Feb 24;13(9):nwag116. doi: 10.1093/nsr/nwag116 (PMC13221956; doi:10.1093/nsr/nwag116)
Supplement: nwag116_Supplemental_File [file nwag116_supplemental_file.pdf]

Supplementary Information for

Molecularly engineered covalent hydrophobic interface for enhanced CO_2 electromethanation in strong acid

Materials and methods

Chemicals and Materials

Copper(II) sulfate hydrate ($CuSO_4 \cdot 5H_2O$) and cysteine (Cys), potassium chloride (KCl), potassium hydroxide (KOH), sulfuric acid (H_2SO_4), polytetrafluoroethylene preparation (PTFE, $(C_2F_4)_n$), fluoroalkyl silane (1H, 1H, 2H, 2H-Perfluorodecyltriethoxysilane, FAS), n-propanol and methanol were purchased from Sinopharm Chemical Reagent Co., Ltd.. Nafion perfluorinated resin solution (5 wt.% in a mixture of lower aliphatic alcohols and water), 3-Trimethylsilyl-1-propane sulfonic acid sodium salt (DSS) were purchased from Tokyo Chemical Industry Co., Ltd.. Deuterium oxide (D_2O) was purchased from Sigma-Aldrich. All chemicals were used without any further purification.

Electrochemical characterization

Electrochemical impedance (EIS) measurement was performed within a frequency range of 100 kHz to 0.01 Hz. The EIS data and DRT analysis were fitted using RelaxIS 3 software (rhd instrument). The electrochemically active surface area (ECSA) of the samples was evaluated by the double layer capacitance (C_{dl}). The C_{dl} was determined by performing cyclic voltammetry (CV) at the potential range of -0.2 to -0.1 V (vs. RHE) at different scan rates (40, 80, 120, 160, and 200 mV s^{-1}). The capacitance currents at -0.15 V vs. RHE were plotted against the scan rates, and the double-layer capacitance (C_{dl}, mF cm^{-2}) was derived from the slope. The kinetic isotopic effect (KIE) experiment was conducted by substituting H_2O with D_2O in the electrolyte.

Catalysts characterization

Scanning electron microscopy (SEM) was conducted using a ZEISS SIGMA500 microscope. Transmission electron microscopy (TEM) and high-resolution TEM (HR-TEM) observations were carried out on a JEOL JEM-2100 instrument operated at 200 kV accelerating voltage. Atomic-resolution characterization was achieved through aberration-corrected high-angle annular dark-field scanning transmission electron microscopy (AC-HAADF-STEM) using a Thermo Fisher Scientific Themis ETEM system. X-ray diffraction (XRD) patterns were recorded on a Rigaku Ultima IV diffractometer with Cu Kα radiation (λ = 1.54056 Å) under operational conditions of 40 kV and 40 mA, employing a scanning rate of 5° min^{-1}. Surface chemical composition was

analyzed by X-ray photoelectron spectroscopy (XPS) using a Quantum 2000 Scanning ESCA Microprobe equipped with monochromatic Al Kα excitation (1486.6 eV). All binding energy values were referenced to the adventitious carbon C 1s peak at 284.8 eV, with spectral deconvolution performed using Gaussian-Lorentzian line shapes following Shirley background subtraction. The contact angle (CA) of water (10 μL) was determined using an OCA 20 instrument (Dataphysics, Germany). Fourier transform infrared (FT-IR) spectroscopic characterization was conducted with a Nicolet 6700 spectrophotometer. X-ray absorption spectroscopy (XAS) was carried out by the BL14W1 station in the Shanghai Synchrotron Radiation Facility (SSRF).

Supporting Figures

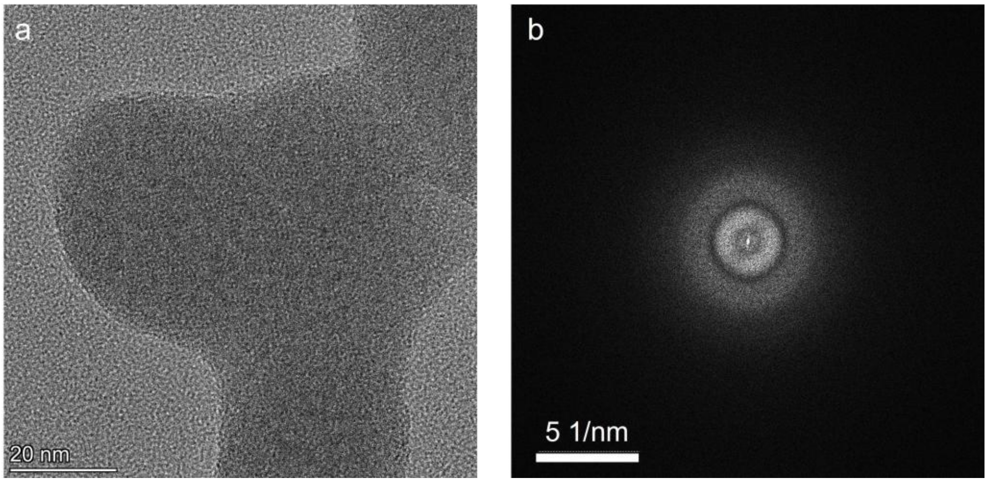

Figure S1 | (a) HR-TEM image and the (b) corresponding FFT diffraction pattern of Cys/Cu.

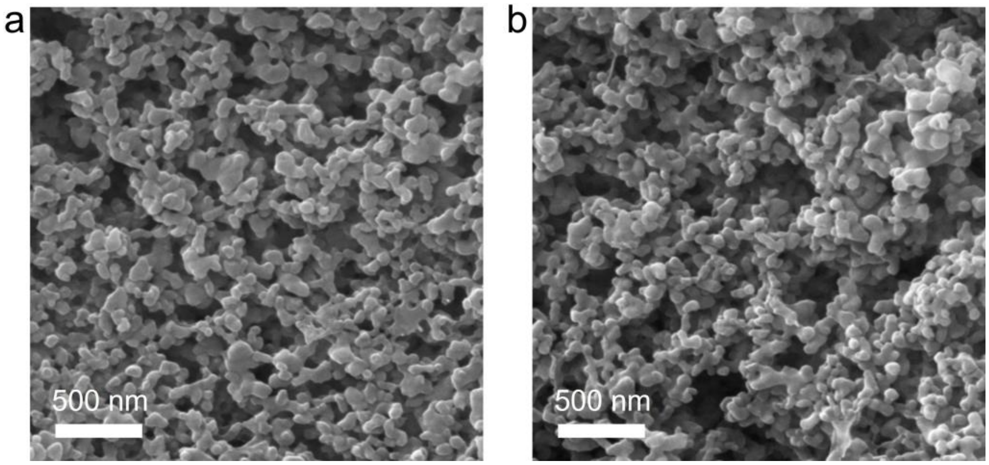

Figure S2 | SEM images of the (a) Cys/Cu and (b) FAS-Cys/Cu-M.

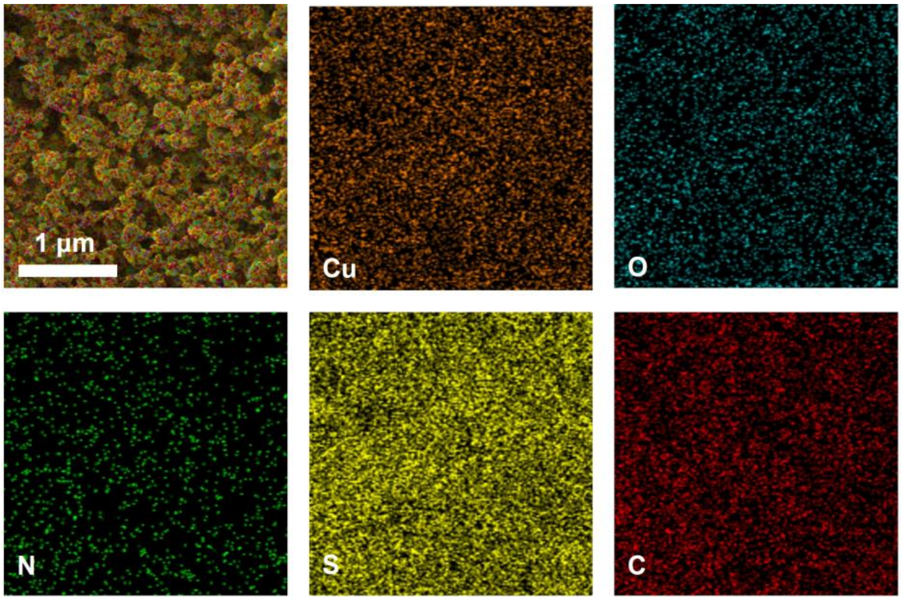

Figure S3 | EDX elemental mapping for Cys/Cu.

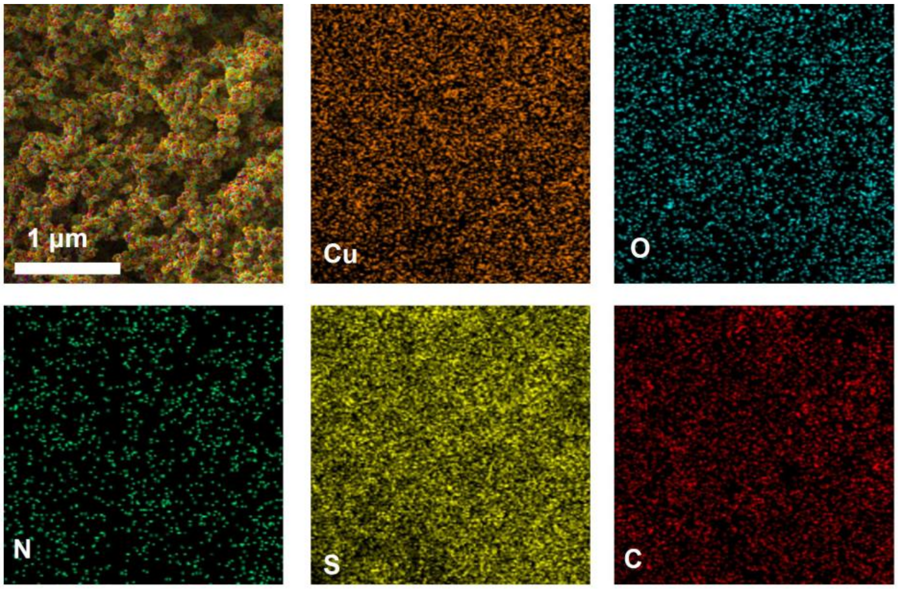

Figure S4 | EDX elemental mapping for FAS-Cys/Cu-M.

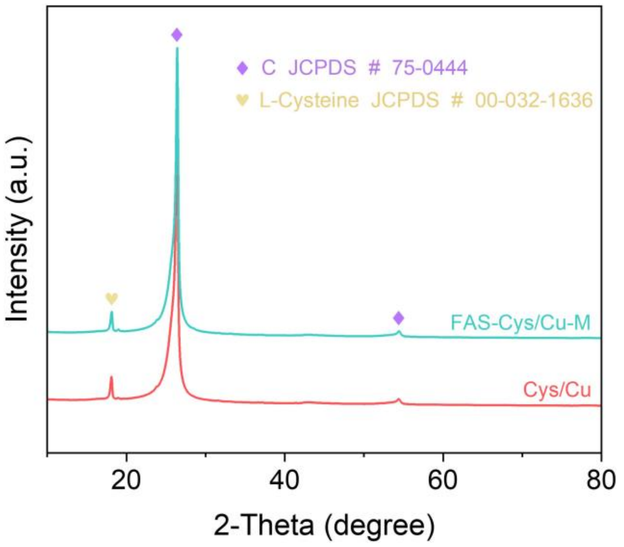

Figure S5 | XRD patterns of the Cys/Cu and FAS-Cys/Cu-M.

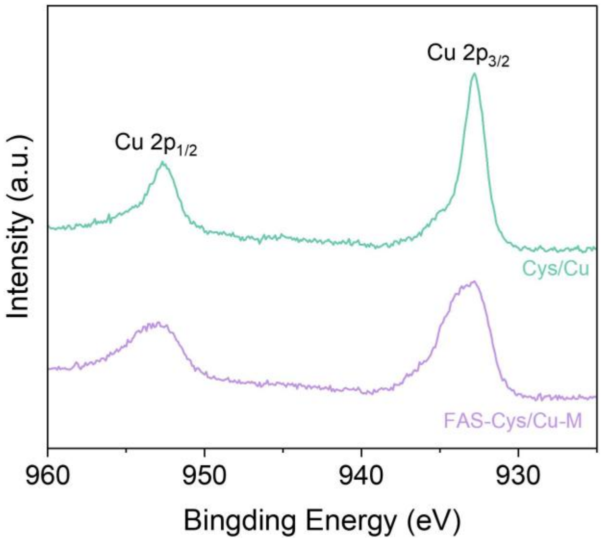

Figure S6 | XPS Cu 2p spectra of Cys/Cu and FAS-Cys/Cu-M.

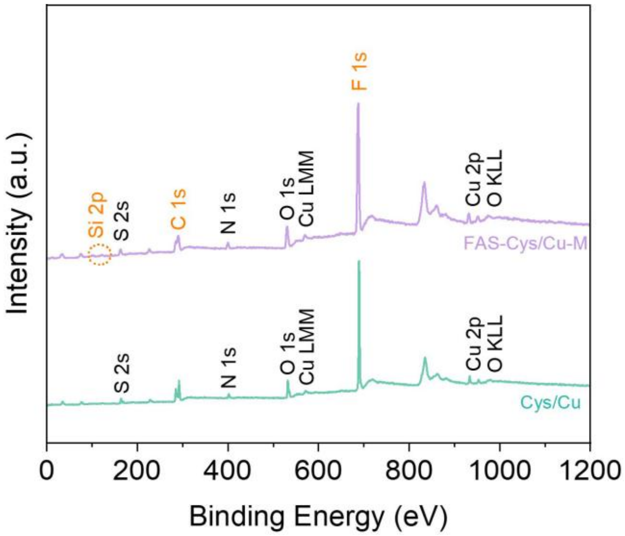

Figure S7 | XPS survey spectra of Cys/Cu and FAS-Cys/Cu-M.

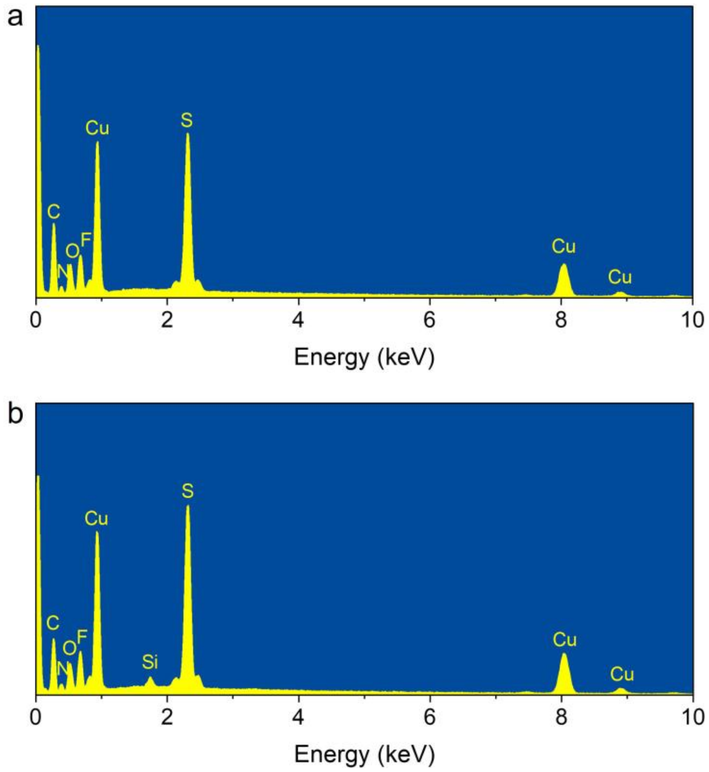

Figure S8 | EDX energy spectra of (a) Cys/Cu and (b) FAS-Cys/Cu-M.

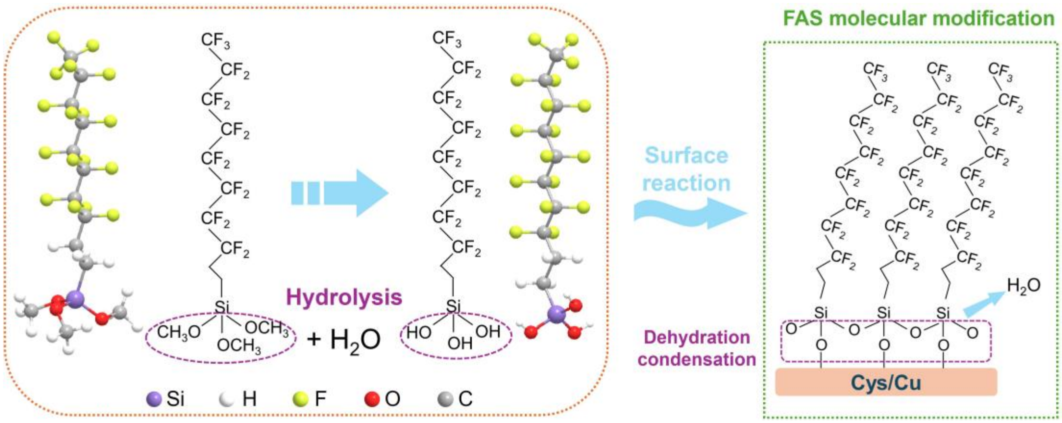

Figure S9 | The schematic diagram for decoration of FAS on the cysteine coated electrode.

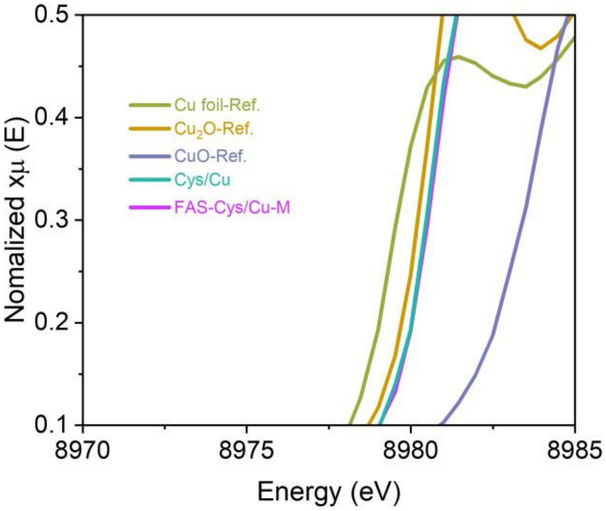

Figure S10 | Cu K-edge XANES spectra for Cys/Cu, FAS-Cys/Cu-M, and reference samples.

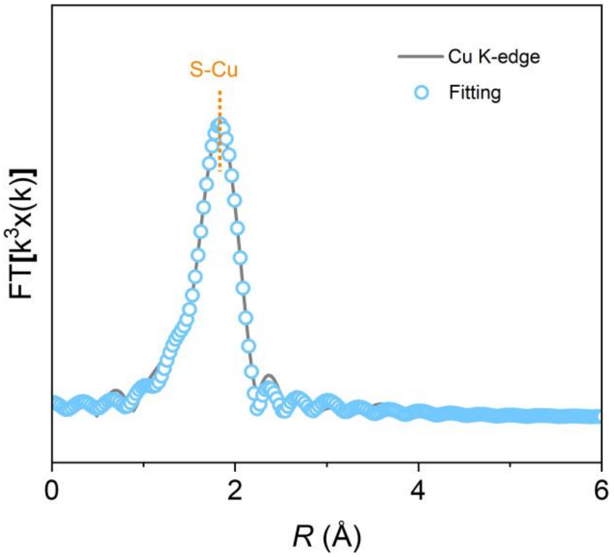

Figure S11 | Experimental and fitting EXAFS curves of FAS-Cys/Cu-M in R space.

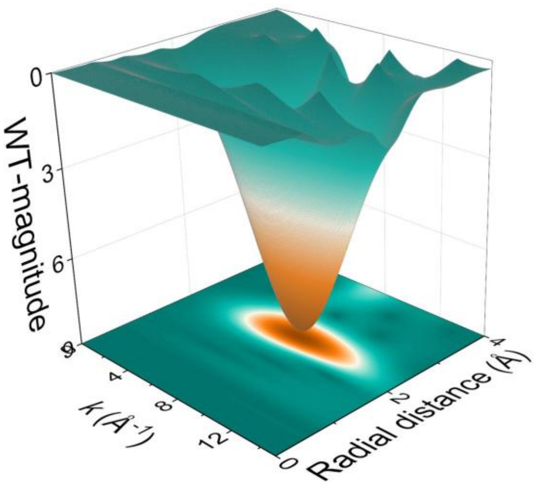

Figure S12 | WT for the k^3-weighted Cu K-edge EXAFS signal of Cu foil.

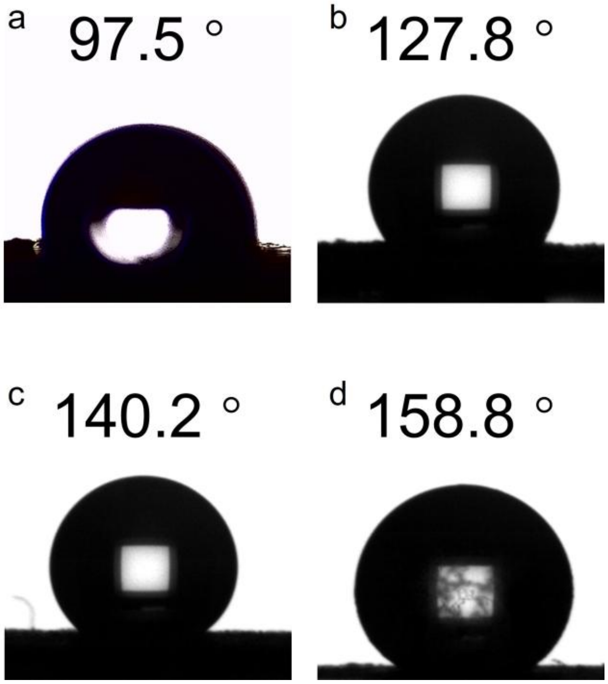

Figure S13 | The water contact angles of (a) Cys/Cu, (b) FAS-Cys/Cu-L, (c) FAS-Cys/Cu-M, and (d) FAS-Cys/Cu-H.

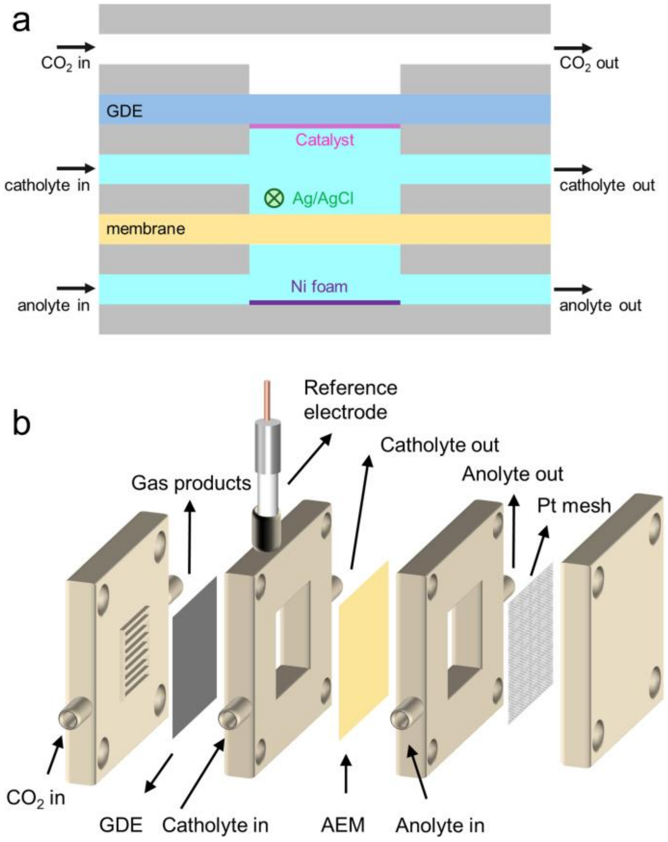

Figure S14 | (a) Schematic illustration and (b) assembly diagram of the flow-cell reactor.

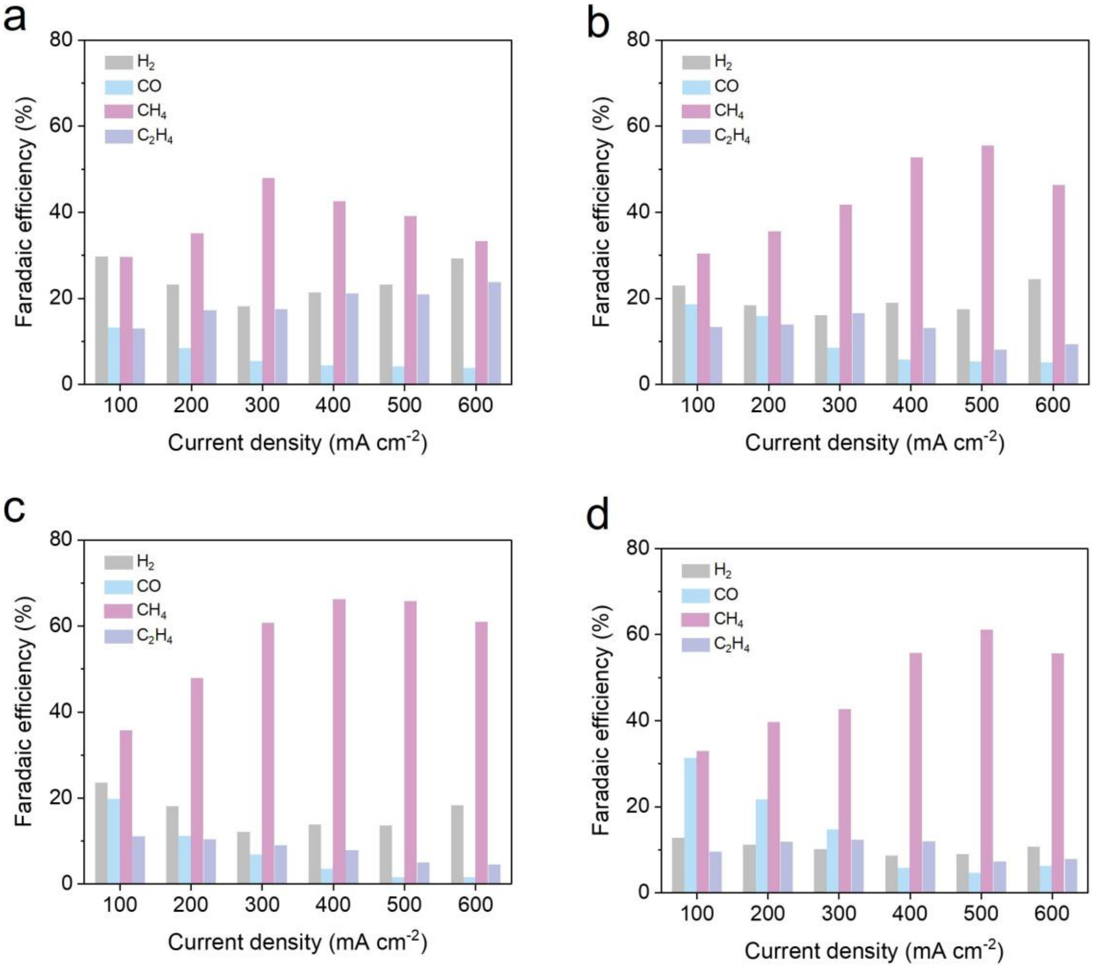

Figure S15 | FEs over (a) Cys/Cu, (b) FAS-Cys/Cu-L, (c) FAS-Cys/Cu-M, and (d) FAS-Cys/Cu-H at a certain current density.

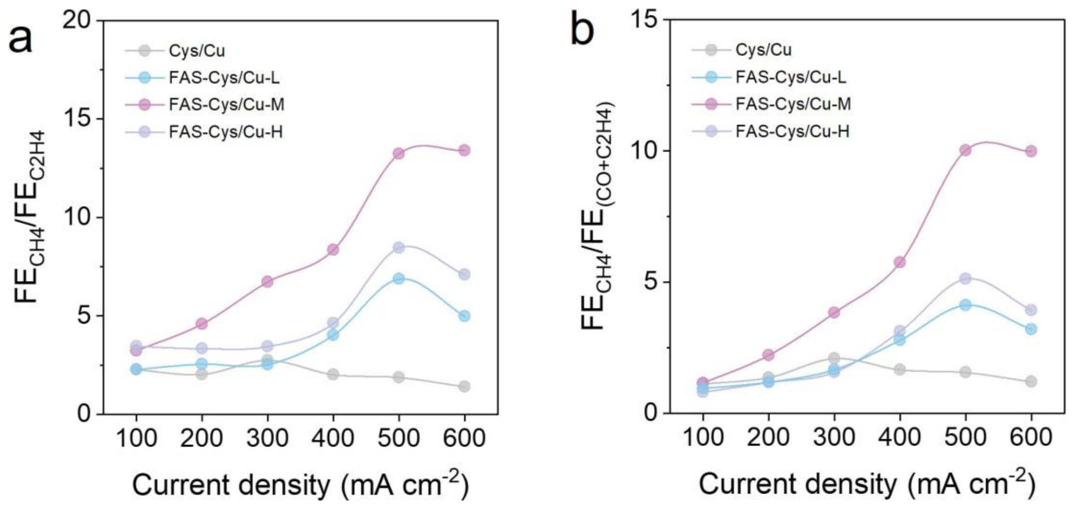

Figure S16 | Ratios of (a) FE_{CH4} to FE_{C2H4}, and (b) FE_{CH4} to $FE_{CO+C2H4}$ for Cys/Cu, FAS-Cys/Cu-L, FAS-Cys/Cu-M, and FAS-Cys/Cu-H.

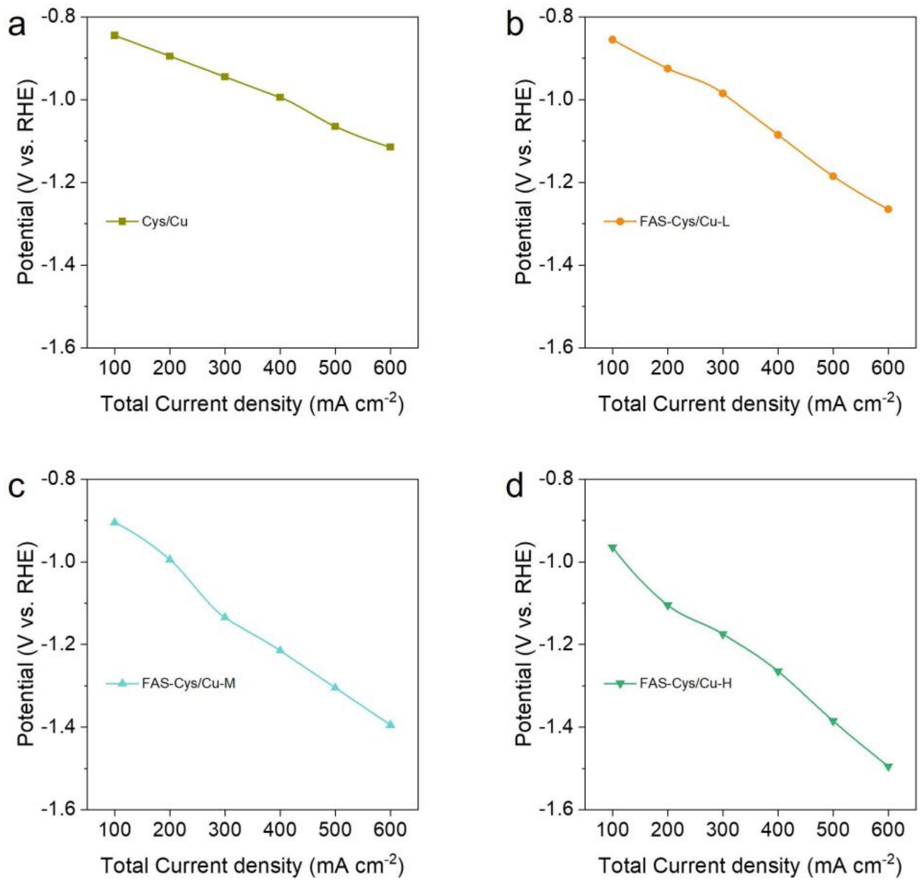

Figure S17 | Potential as a function of current density over (a) Cys/Cu, (b) FAS-Cys/Cu-L, (c) FAS-Cys/Cu-M, and (d) FAS-Cys/Cu-H.

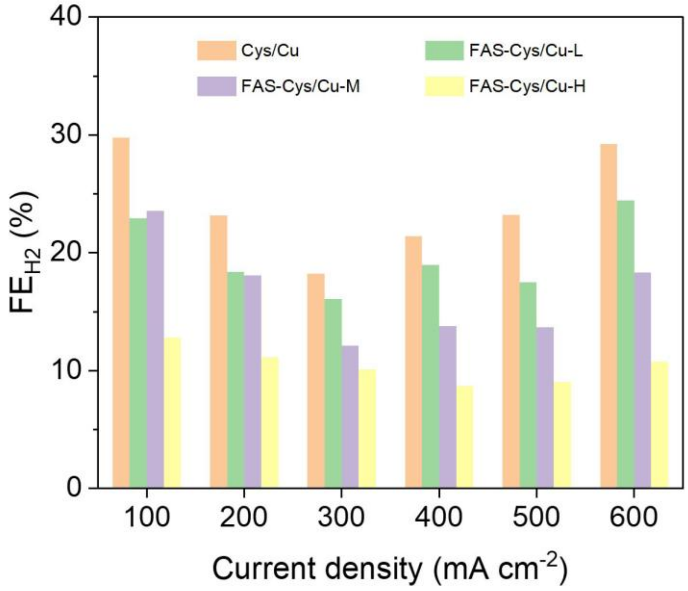

Figure S18 | The H_2 FE for Cys/Cu, FAS-Cys/Cu-L, FAS-Cys/Cu-M, and FAS-Cys/Cu-H.

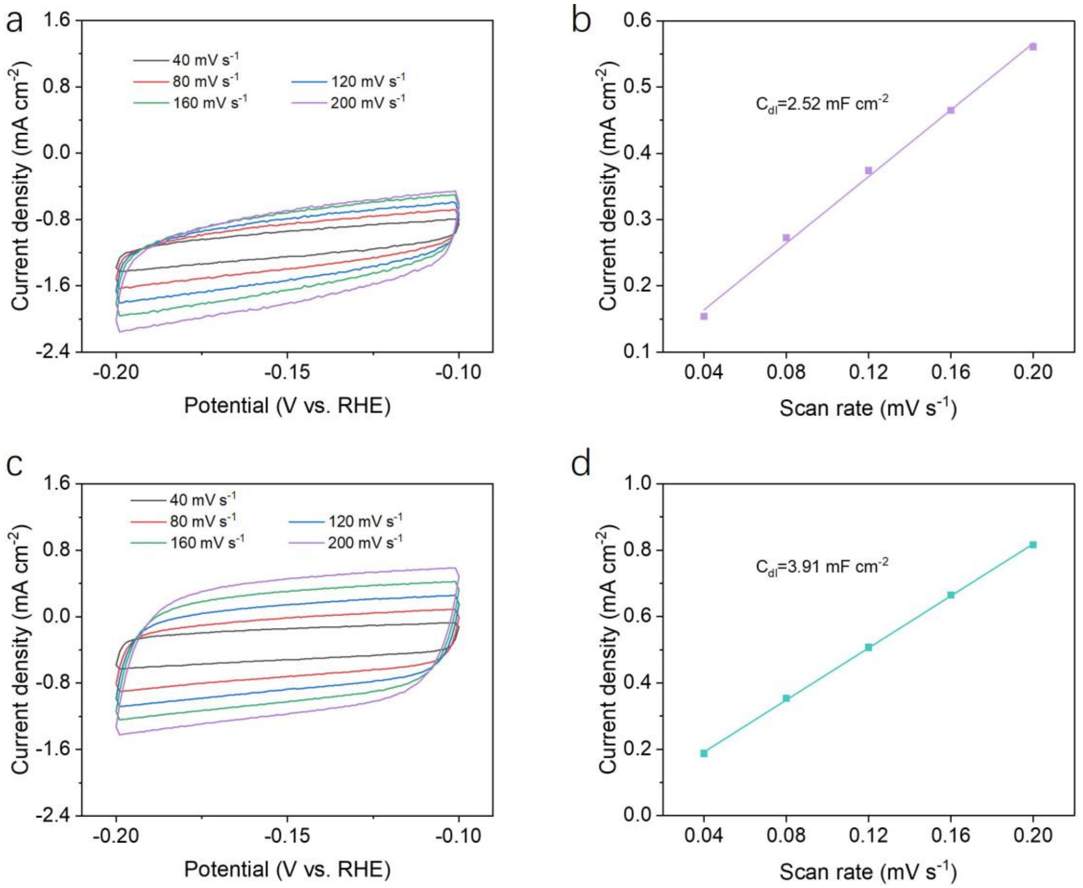

Figure S19 | Cyclic voltammetry curves of (a) FAS-Cys/Cu-M and (c) Cys/Cu. The plot of current density against the scan rates of (b) FAS-Cys/Cu-M and (d) Cys/Cu.

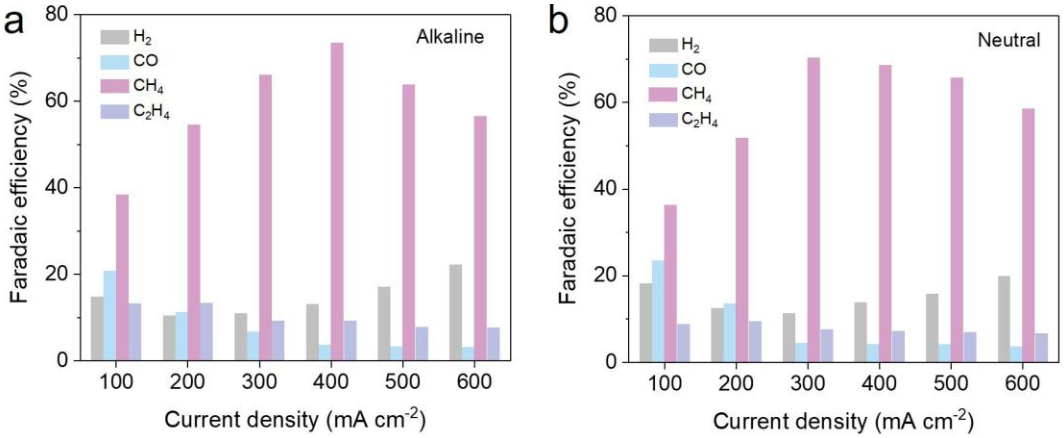

Figure S20 | Gas products FEs of CO_2ERR over a FAS-Cys/Cu-M in (a) alkaline electrolyte (1.0 M KOH) and (b) neutral electrolyte (1.0 M $KHCO_3$).

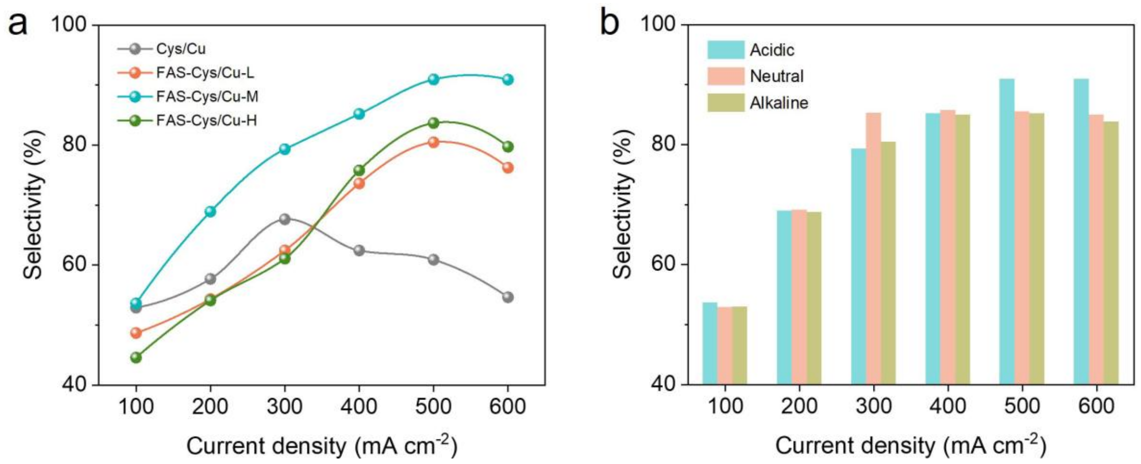

Figure S21 | (a) Selectivity of CH_4 in gaseous CO_2ERR products at different current densities on Cys/Cu, FAS-Cys/Cu-L, FAS-Cys/Cu-M, and FAS-Cys/Cu-H. (b) Selectivity of CH_4 in gaseous products at different current densities of FAS-Cys/Cu-M in various pH electrolytes.

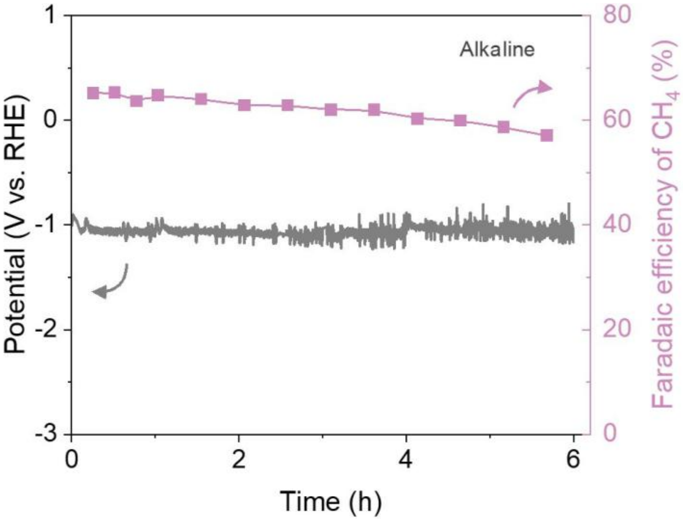

Figure S22 | Long-term CO_2ERR stability of FAS-Cys/Cu-M in alkaline electrolyte.

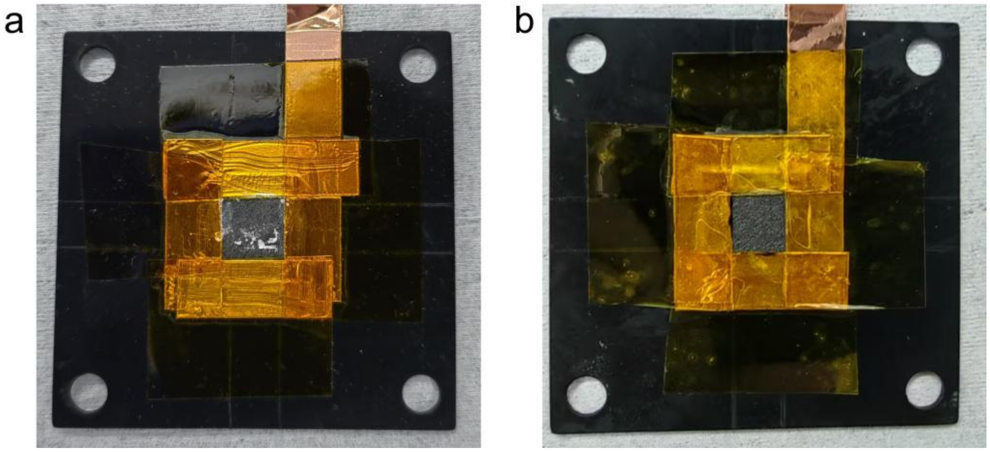

Figure S23 | Photos of FAS-Cys/Cu-M after electrolysis in (a) alkaline and (b) acidic electrolytes.

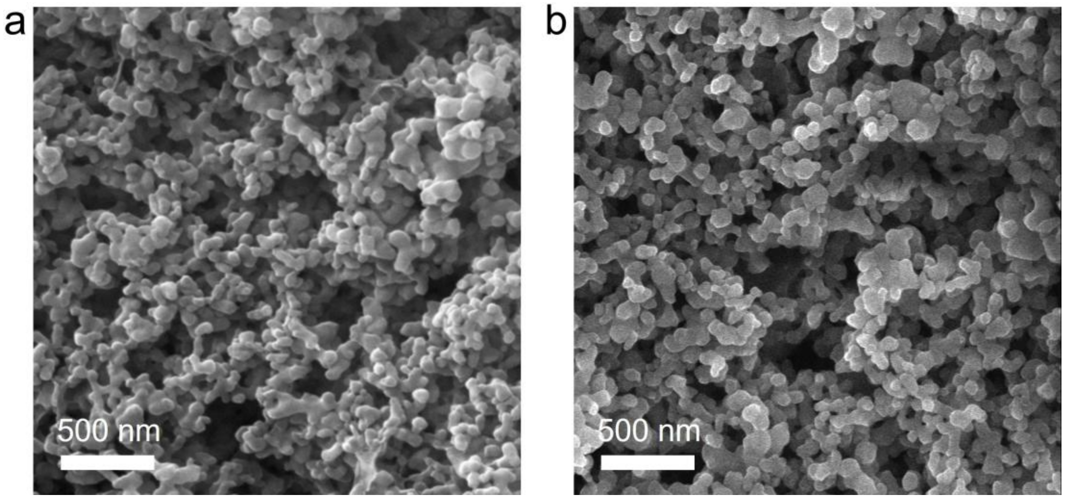

Figure S24 | SEM images of FAS-Cys/Cu-M (a) before reaction and (b) after reaction.

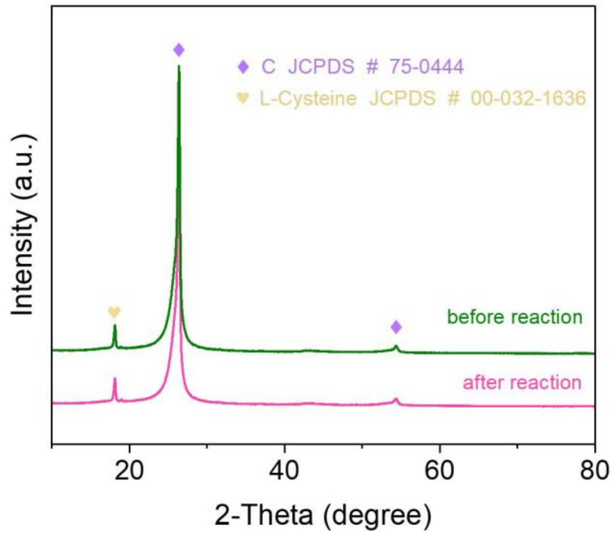

Figure S25 | XRD patterns of FAS-Cys/Cu-M before and after reactions.

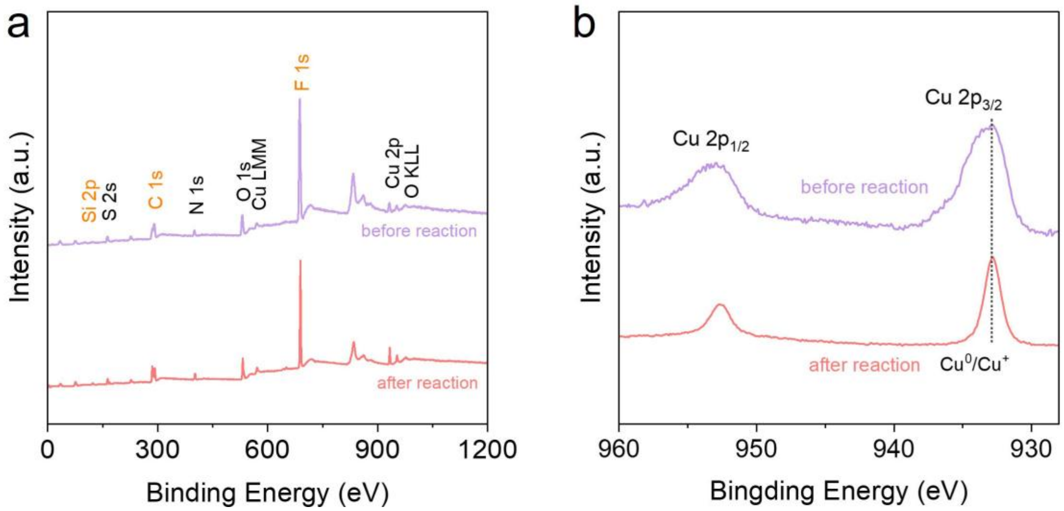

Figure S26 | (a) XPS survey spectra and (b) Cu 2p spectra of FAS-Cys/Cu-M before and after the reactions.

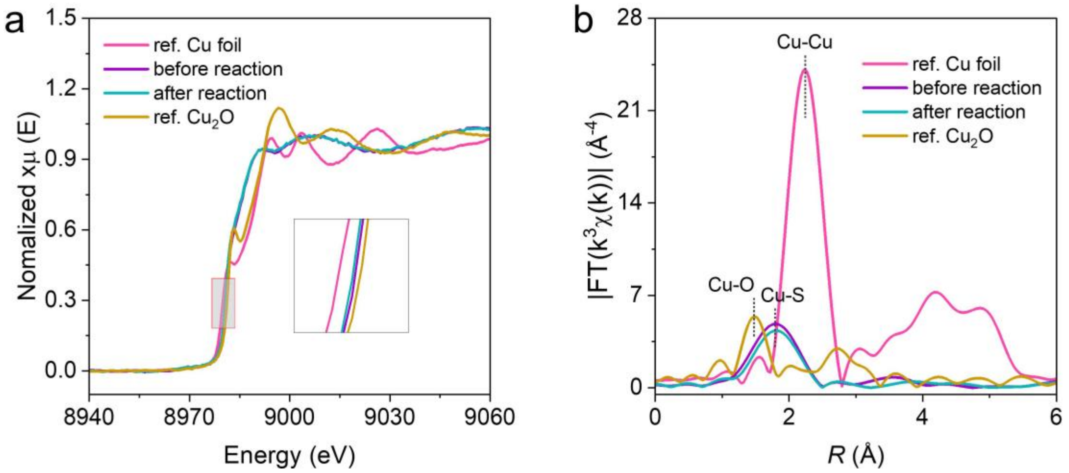

Figure S27 | (a) Cu K-edge XANES and (b) FT-EXAFS spectra before and after the reactions of FAS-Cys/Cu-M.

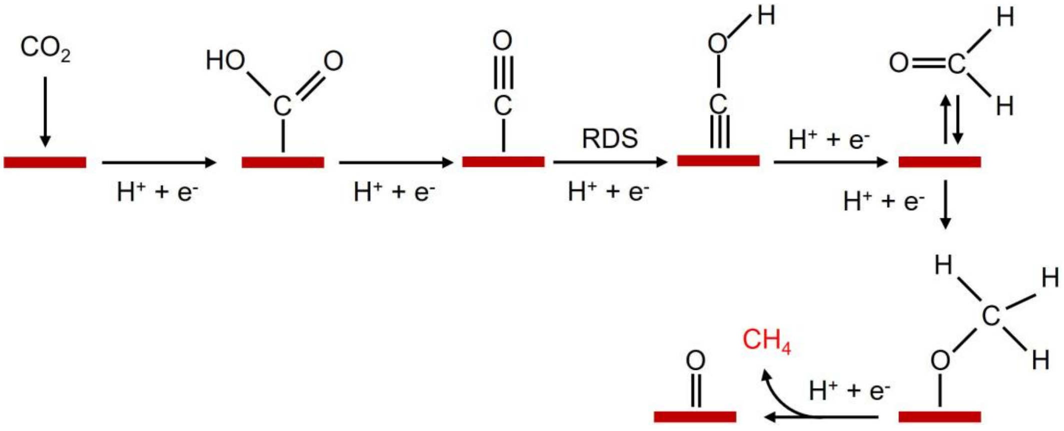

Figure S28 | Proposed reaction pathway for CO_2 to CH_4 conversion.

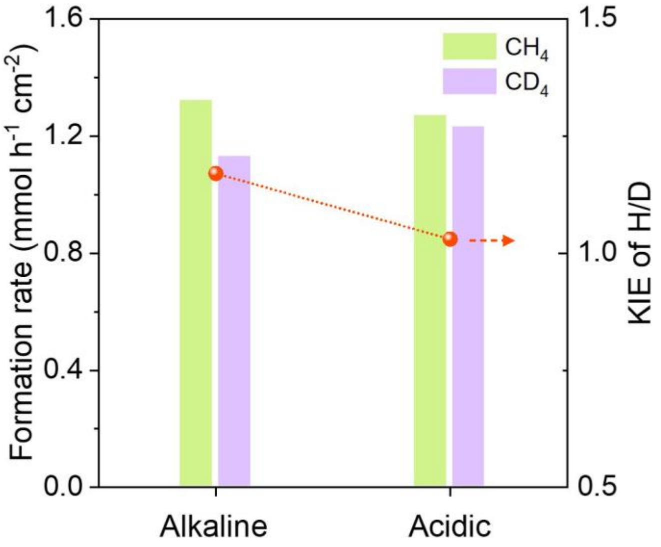

Figure S29 | The formation rates (CH_4 and CD_4) and KIE of H/D of FAS-Cys/Cu-M at 400 mA cm^{-2}.

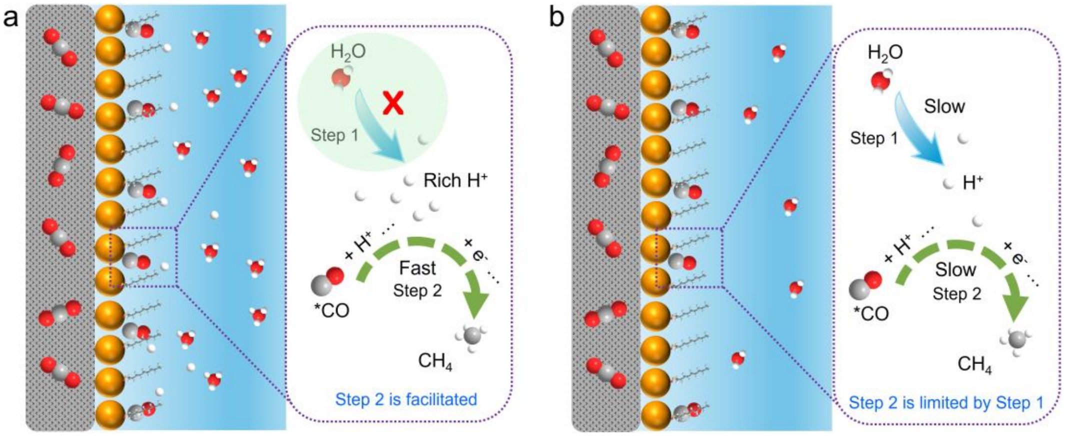

Figure S30 | Schematic diagram of the mechanism of *CO hydrogenation to CH_4 on the surface of FAS modified electrodes in (a) acidic and (b) alkaline electrolytes.

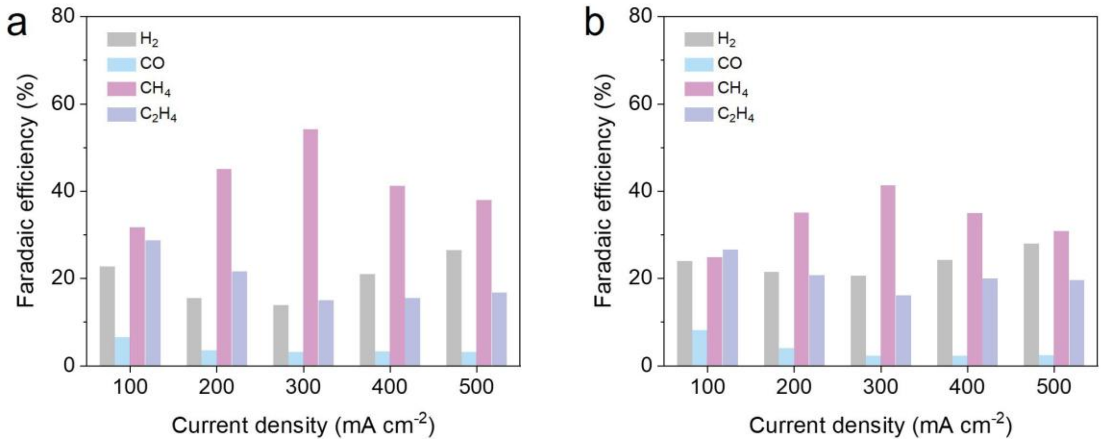

Figure S31 | Gas products FEs of CO_2ERR over (a) PTFE-Cys/Cu-L and (b) PTFE-Cys/Cu-H.

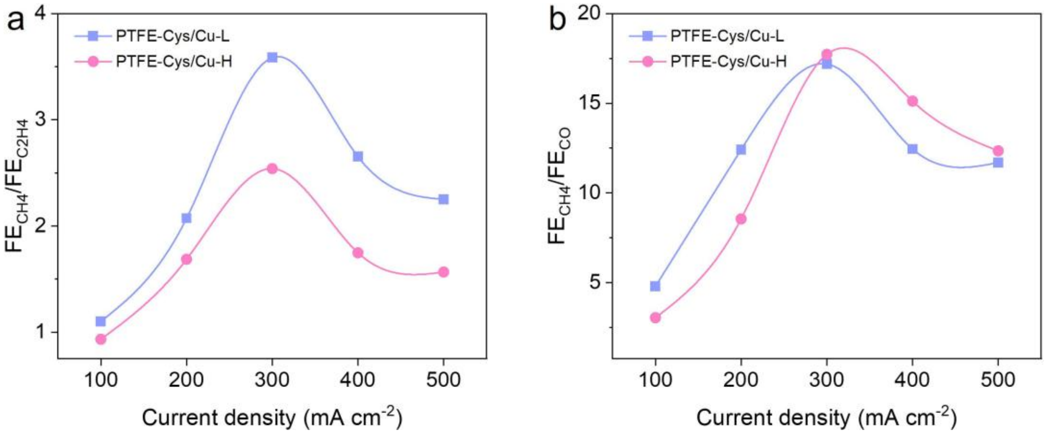

Figure S32 | (a) Ratios of FE_{CH4} to FE_{C2H4} and (b) ratios of FE_{CH4} to FE_{CO} at different current densities.

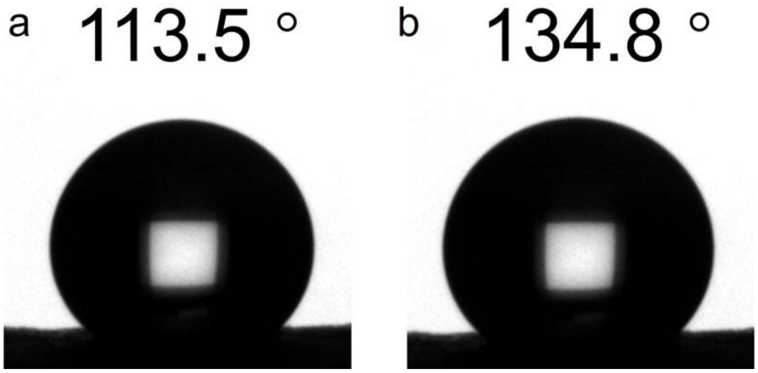

Figure S33 | The water contact angles of (a) PTFE-Cys/Cu-L and (b) PTFE-Cys/Cu-H.

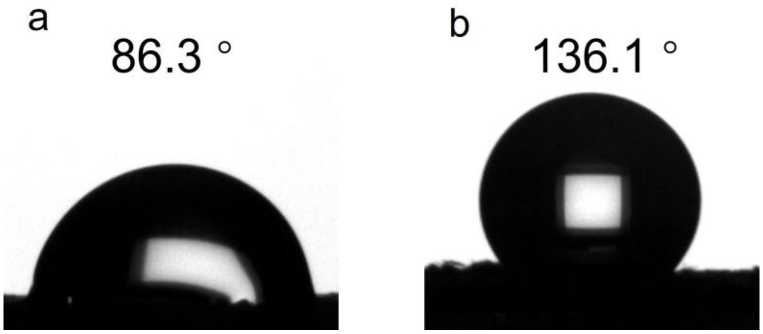

Figure S34 | The water contact angles measured after electrolysis for (a) PTFE-Cys/Cu-L and (b) FAS-Cys/Cu-M.

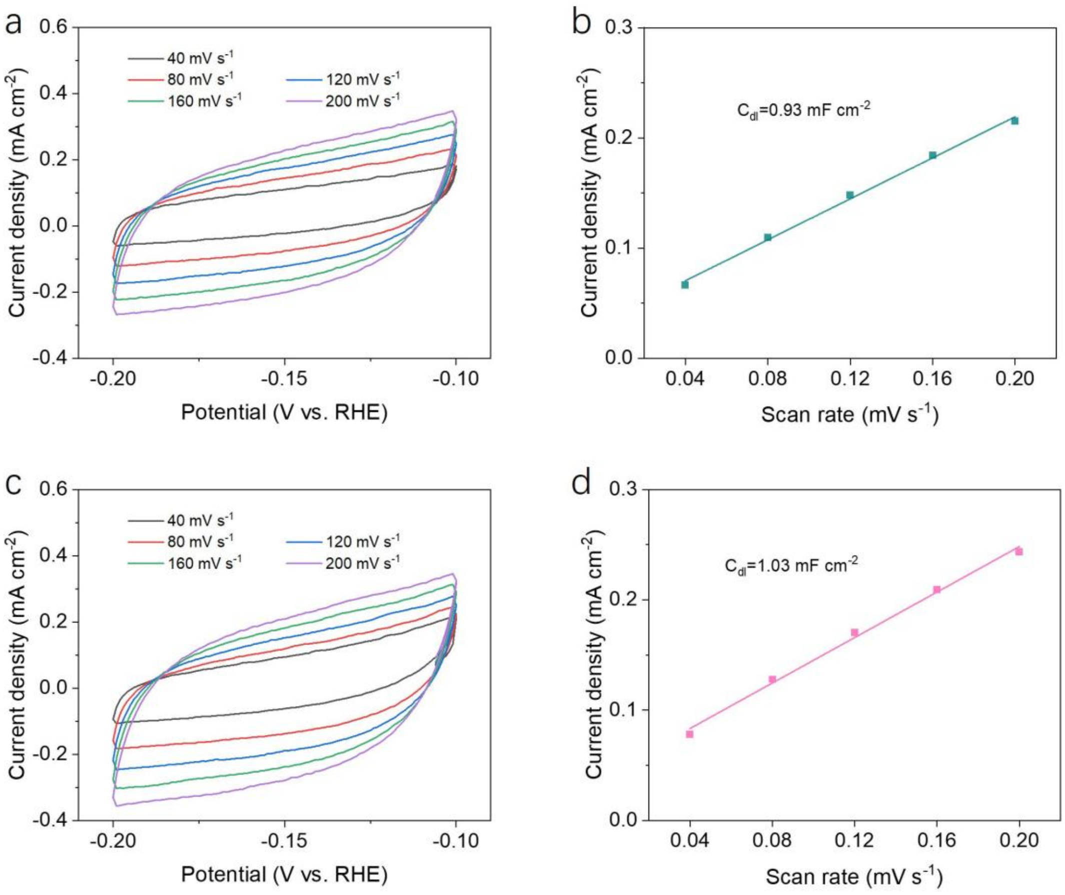

Figure S35 | Cyclic voltammetry curves of (a) PTFE-Cys/Cu-L and (c) PTFE-Cys/Cu-H. The plot of current density against the scan rates of (b) PTFE-Cys/Cu-L and (d) PTFE-Cys/Cu-H.

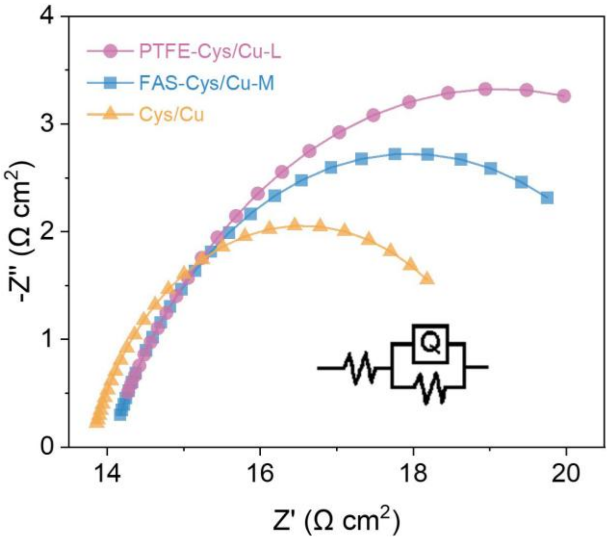

Figure S36 | Nyquist plots of PTFE-Cys/Cu-L, FAS-Cys/Cu-M, and Cys/Cu, the inset shows the equivalent circuit diagram.

Supporting Tables

Table S1 | Extended X-ray absorption fine structure (EXAFS) fitting parameters.

Sample	Coordination	*N*	*R* (Å)	ΔE_0 (eV)	σ^2 (10^{-3}Å^2)	*R* factor
Cu foil	Cu−Cu	12	2.56 ± 0.02	4.3 ± 0.6	8.4	0.005
Cys/Cu	Cu−S	2.7 ± 0.2	2.30 ± 0.05	2.9 ± 0.8	6.8	0.002
FAS-Cys/Cu-M	Cu−S	2.5 ± 0.2	2.30 ± 0.06	2.5 ± 0.9	6.8	0.002

Table S2 | pH values of different cathode electrolytes.

Electrolyte	First	Second	Third	Average
1.0 M KOH	13.38	13.52	13.46	13.45
1.0 M $KHCO_3$	8.33	8.27	8.31	8.30
3.0 M KCl with H_2SO_4	1.81	1.79	1.78	1.79

Table S3 | pH recording of cathode solutions during CO_2ERR.

**Reaction time (h)**	**Catholyte pH**
0	1.79
1	1.82
2	1.88
3	1.86
4	1.89
5	1.84
6	1.88
7	1.91
8	2.02
9	2.11
10	2.15

Table S4 | The amount of dissolved Cu in the outlet electrolyte quantified by ICP-OES analysis.

**Reaction time (h)**	**Dissolved Cu**
0	Not detected
0.5	Not detected
1.0	Not detected
1.5	Not detected
2.0	Not detected

Table S5 | Performance comparison with previously reported CO_2ERR systems.

**Catalyst**	**Potential (V vs. RHE)**	**Electrolyte**	**FE_{CH4} (%)**	**j_{CH4} (mA cm^{-2})**	**Ref.**
**FAS-Cys/Cu-M**	−1.21	3.0 M KCl + H_2SO_4	66.2	264.7	This work
**FAS-Cys/Cu-M**	−0.96	1.0 M $KHCO_3$	70.4	211.1	
**FAS-Cys/Cu-M**	−0.90	1.0 M KOH	73.5	293.8	
**Cu-18-C-6**	/	0.5 M K_2SO_4 + H_2SO_4	51.2	307.1	[1]
**Cu-Ce-O_x**	−0.82	1.0 M KOH	67.8	135.6	[2]
**Cu/La_2CuO_4**	−1.40	1.0 M KOH	56.3	116.5	[3]
**Cu/Al_2O_3**	−1.20	1.0 M KOH	62.0	94.9	[4]
**Cu-N/IPCF**	−1.21	1.0 M KOH	74.2	74.2	[5]
**Sr_2CuWO_6**	−1.15	1.0 M KOH	73.1	73.1	[6]
**Cu-SAs/HGDY**	−1.10	1.0 M KOH	72.1	72.1	[7]
**La_5Cu_{95}**	−1.72	1.0 M KOH	64.5	64.5	[8]
**CuSiOx**	−1.60	1.0 M KOH	55.3	55.3	[9]
**Cu-PzI**	−1.00	1.0 M KOH	52.0	52.0	[10]
**N-aGQDs-A9**	−1.04	1.0 M KOH	46.0	46.0	[11]
**CoO/Cu**	−1.11	1.0 M $KHCO_3$	60.0	135.0	[12]
**Au/Cu**	−1.23	1.0 M $KHCO_3$	56.0	112.0	[13]
**Cu**	−1.41	1.0 M $KHCO_3$	48.0	108.0	[14]
**CuPc**	−1.06	1.0 M $KHCO_3$	66.0	8.6	[15]
**Cu/CeO_2**	−1.80	0.1 M $KHCO_3$	58.0	40.6	[16]
**Cu-N-C**	−1.60	0.1 M $KHCO_3$	38.6	14.8	[17]
**Cu_2O/Cu-MOF**	−1.71	0.1 M $KHCO_3$	63.2	8.4	[18]

Table S6 | Compared with the other recently reported typical catalyst for CO_2ERR.

Catalyst	Electrolyte	pH	SPCE (%)	FE (%)	j_{CH4} (mA cm^{-2})	Yield (mmol h^{-1} cm^{-2})	Stability (h)	Ref.
**FAS-Cys/Cu-M**	3.0 M KCl+M H_2SO_4	1.8	31.1 at 2 sccm	66.2	264.7	1.24	10	This work
**Cu-18-C-6**	0.5 M K_2SO_4+H_2SO_4	2.0	28.2 at 2 sccm	51.2	307.1	1.43	4.4	[1]
**Co Terpy-RF**	0.5 M $KHCO_3$	7.2	0.2 at 10 sccm	80.0	8.6	0.04	9	[19]
**CoO/Cu**	1.0 M $KHCO_3$	7.8	2.4 at 10 sccm	60.0	135.0	0.63	18	[12]
**Sr_2CuWO_6**	1.0 M KOH	14.0	1.5 at 35 sccm	73.1	292.4	1.36	5.5	[6]
**Cu-SAs/HGDY**	1.0 M KOH	14.0	1.3 at 30 sccm	72.1	230.7	1.08	11	[7]

References

1. Xu K, Li J, Liu F, Chen X, Zhao T, Cheng F. Favoring CO Intermediate Stabilization and Protonation by Crown Ether for CO_2 Electromethanation in Acidic Media. *Angew. Chem. Int. Ed.* 2023: e202311968.
2. Zhou X, Shan J, Chen L, et al. Stabilizing Cu^{2+} Ions by Solid Solutions to Promote CO_2 Electroreduction to Methane. *J. Am. Chem. Soc.* 2022; 144: 2079-2084.
3. Chen S, Su Y, Deng P, et al. Highly Selective Carbon Dioxide Electroreduction on Structure-Evolved Copper Perovskite Oxide toward Methane Production. *ACS Catal.* 2020; 10: 4640-4646.
4. Chen S, Wang B, Zhu J, et al. Lewis Acid Site-Promoted Single-Atomic Cu Catalyzes Electrochemical CO_2 Methanation. *Nano Lett.* 2021; 21: 7325-7331.
5. Pan F, Fang L, Li B, et al. N and OH-Immobilized Cu_3 Clusters In Situ Reconstructed from Single-Metal Sites for Efficient CO_2 Electromethanation in Bicontinuous Mesochannels. *J. Am. Chem. Soc.* 2024; 146: 1423-1434.
6. Zhu J, Zhang Y, Chen Z, et al. Superexchange-stabilized long-distance Cu sites in rock-salt-ordered double perovskite oxides for CO_2 electromethanation. *Nat. Commun.* 2024; 15: 1565.
7. Zhao P, Jiang H, Shen H, et al. Construction of Low-Coordination Cu-C_2 Single-Atoms Electrocatalyst Facilitating the Efficient Electrochemical CO_2 Reduction to Methane. *Angew. Chem. Int. Ed.* 2023: e202314121.
8. Zhao J, Zhang P, Yuan T, et al. Modulation of *CH_xO Adsorption to Facilitate Electrocatalytic Reduction of CO_2 to CH_4 over Cu-Based Catalysts. *J. Am. Chem. Soc.* 2023; 145: 6622-6627.
9. Tan X, Sun K, Zhuang Z, et al. Stabilizing Copper by a Reconstruction-Resistant Atomic Cu-O-Si Interface for Electrochemical CO_2 Reduction. *J. Am. Chem. Soc.* 2023; 145: 6622-6627.
10. Wang R, Liu J, Huang Q, Dong LZ, Li SL, Lan YQ. Partial Coordination-Perturbed Bi-Copper Sites for Selective Electroreduction of CO_2 to Hydrocarbons. *Angew. Chem. Int. Ed.* 2021; 60: 19829-19835.
11. Yadav RM, Li Z, Zhang T, et al. Amine-Functionalized Carbon Nanodot Electrocatalysts Converting Carbon Dioxide to Methane. *Adv. Mater.* 2021; 24: e2105690.

12. Li Y, Xu A, Lum Y, et al. Promoting CO_2 methanation via ligand-stabilized metal oxide clusters as hydrogen-donating motifs. *Nat. Commun.* 2020; 11: 6190.
13. Wang X, Ou P, Wicks J, et al. Gold-in-copper at low *CO coverage enables efficient electromethanation of CO_2. *Nat. Commun.* 2021; 12: 3387.
14. Wang X, Xu A, Li F, et al. Efficient Methane Electrosynthesis Enabled by Tuning Local CO_2 Availability. *J. Am. Chem. Soc.* 2020; 142: 3525-3531.
15. Weng Z, Wu Y, Wang M, et al. Active sites of copper-complex catalytic materials for electrochemical carbon dioxide reduction. *Nat. Commun.* 2018; 9: 415.
16. Wang Y, Chen Z, Han P, et al. Single-Atomic Cu with Multiple Oxygen Vacancies on Ceria for Electrocatalytic CO_2 Reduction to CH_4. *ACS Catal.* 2018; 8: 7113-7119.
17. Guan A, Chen Z, Quan Y, et al. Boosting CO_2 Electroreduction to CH4 via Tuning Neighboring Single-Copper Sites. *ACS Energy Lett.* 2020; 5: 1044-1053.
18. Tan X, Yu C, Zhao C, et al. Restructuring of Cu_2O to Cu_2O@Cu-Metal–Organic Frameworks for Selective Electrochemical Reduction of CO_2. *ACS Appl. Mater. Interfaces* 2019; 11:9904-9910.
19. McKee M, Kutter M, Wu Y, et al. Hydrophobic assembly of molecular catalysts at the gas–liquid–solid interface drives highly selective CO_2 electromethanation. *Nat. Chem.* 2024; 17: 92-100.
